# Intrinsic response of thoracic propriospinal neurons to axotomy

**DOI:** 10.1186/1471-2202-11-69

**Published:** 2010-06-04

**Authors:** Justin R Siebert, Frank A Middelton, Dennis J Stelzner

**Affiliations:** 1 Department of Cell and Developmental Biology, SUNY Upstate Medical University, 750 East Adams Street, Syracuse New York, USA; 2 Department of Neuroscience and Physiology, SUNY Upstate Medical University, 750 East Adams Street, Syracuse New York, USA

## Abstract

**Background:**

Central nervous system axons lack a robust regenerative response following spinal cord injury (SCI) and regeneration is usually abortive. Supraspinal pathways, which are the most commonly studied for their regenerative potential, demonstrate a limited regenerative ability. On the other hand, propriospinal (PS) neurons, with axons intrinsic to the spinal cord, have shown a greater regenerative response than their supraspinal counterparts, but remain relatively understudied in regards to spinal cord injury.

**Results:**

Utilizing laser microdissection, gene-microarray, qRT-PCR, and immunohistochemistry, we focused on the intrinsic post-axotomy response of specifically labelled thoracic propriospinal neurons at periods from 3-days to 1-month following T9 spinal cord injury. We found a strong and early (3-days post injury, p.i) upregulation in the expression of genes involved in the immune/inflammatory response that returned towards normal by 1-week p.i. In addition, several regeneration associated and cell survival/neuroprotective genes were significantly up-regulated at the earliest p.i. period studied. Significant upregulation of several growth factor receptor genes (GFRa1, Ret, Lifr) also occurred only during the initial period examined. The expression of a number of pro-apoptotic genes up-regulated at 3-days p.i. suggest that changes in gene expression after this period may have resulted from analyzing surviving TPS neurons after the cell death of the remainder of the axotomized TPS neuronal population.

**Conclusions:**

Taken collectively these data demonstrate that thoracic propriospinal (TPS) neurons mount a very dynamic response following low thoracic axotomy that includes a strong regenerative response, but also results in the cell death of many axotomized TPS neurons in the first week after spinal cord injury. These data also suggest that the immune/inflammatory response may have an important role in mediating the early strong regenerative response, as well as the apoptotic response, since expression of all of three classes of gene are up-regulated only during the initial period examined, 3-days post-SCI. The up-regulation in the expression of genes for several growth factor receptors during the first week post-SCI also suggest that administration of these factors may protect TPS neurons from cell death and maintain a regenerative response, but only if given during the early period after injury.

## Background

Spinal cord injury (SCI) creates an environment enriched in factors that inhibit axonal regeneration including myelin proteins and chondroitin sulfate proteoglycans that has been extensively reviewed [1-3]. The supraspinal pathways most often tested after SCI for their regenerative ability (i.e. corticospinal tract; rubrospinal tract), have limited inherent regenerative abilities, even when more permissive environments are established at the injury site [4,5]. One factor related to the limited regeneration of supraspinal axons is the long distance between the site of axotomy and their cell bodies of origin. Previous studies have demonstrated neurons mount a stronger regenerative response if axotomy occurs closer to the cell body [6-8]. This may be why propriospinal (PS) axons, from neurons intrinsic to the spinal cord, show greater regenerative ability than supraspinal nerve tracts when the same experimental strategies are used to increase regeneration after SCI [9-13].

In concert with supraspinal nerve tracts, PS neurons play an important role in locomotor function, limb coordination, and postural support [14]. In spite of their importance in spinal motor function, as well as their greater regenerative potential, and potential for post-injury axonal plasticity [15,16], PS neurons have been relatively understudied. Preliminary studies from our laboratory show an initial large loss of thoracic PS (TPS) neurons two weeks following moderate T9-10 spinal contusion injury (25 mm weight drop, NYU Impactor), but ~30% of axotomized TPS neurons remain rostral to this injury for at least one month, as assessed by prelabeling these cells from the lumbosacral spinal cord prior to spinal injury [17]. The present investigation is a detailed analysis of the cellular response of TPS neurons after low thoracic complete SCI. We paid particular attention to changes related to their regenerative ability and to early cell loss, based on the hypothesis that the short distance from the lesion would ensure a maximal regenerative response.

Using whole-transcriptome profiling with microarrays, followed by qRT-PCR and immunohistochemistry for validation, we found a strong initial inflammatory response as well as an early regeneration and cell death response. An up-regulation of several growth factor receptors, as well as a down-regulation of receptors to several factors that inhibit axonal growth may indicate potential therapeutic treatments to protect TPS neurons from early cell death post-axotomy and to maximize and sustain the early regenerative response.

## Methods

The SUNY Upstate Medical University Committee for the Humane Use of Animals, following the provisions and guidelines set forth by the institutional Department of Laboratory Animal Research and the Association for Assessment and Accreditation of Laboratory Animal Care (A.A.A.L.A.C), approved all procedures involving the use of animals.

Female hooded Long-Evans rats (N = 36, Simonsen; Santa Clara; CA) approximately 77 days (± 10 days) old were used in this study. Animals were assigned to various labeling, injury, and survival time-points (see Table 1 for group assignments).

**Table 1:** Animal and Experimental Treatment Groups

Animal Group	N	Label	Injury	Type	Survival Post Injury	Total Survival Time Post Label	Group Comparison
1	4	FG	No	---	---	10 Days	1 to 4
2	4	FG	No	---	---	3 Weeks	1 to 5
3	4	FG	No	---	---	5 Weeks	2 to 6
4	4	FG	Yes	TXN	3 Days	10 Days	3 to 7
5	4	FG	Yes	TXN	1 Week	2 Weeks	
6	4	FG	Yes	TXN	2 Weeks	3 Weeks	
7	4	FG	Yes	TXN	5 Weeks	5 Weeks	
8	4	DTMR	Yes	CON	1 Week	2 Weeks	8 to 9
9	4	DTMR	No	---	---	2 weeks	8 to 5

Female Long-Evans rat were divided among 9 groups. Then using the indicated tracer, Fluorogold (FG) or Dextran Tetramethyl Rhodamine (DTMR), TPS neurons were prelabeled by a series of bilateral injections into the upper lumbrosacral enlargement. Following 1-week tracer transport time, animals in groups 4-8 were subjected to either a T9 complete spinal transection (TXN) or moderate spinal contusion injury (25 mm weight drop, NYU Impactor; CON). Animals were then allowed to recover for different periods post-injury as indicated. N used in analysis indicates the number of animals that were used in the data analysis part of this study. (*) Group 6 only has an N of 2 due to 1 animal failing to pass the microarray's internal quality control, and the loss of 1 animal to freezing artifact during histological processing. (*) Group 7 only has an N of 2 due to premature animal mortality. The final column of this table indicates the time point comparisons that were made for both the gene and PCR validation studies conducted during this study.

### Animal Surgeries

#### Retrograde Labelling of Thoracic Propriospinal (TPS) Neurons

Animals were anesthetized by an intraperitoneal (IP) injection of a ketamine/xylazine cocktail (0.07 cc/100 g). Once the animal was areflexic, a laminectomy was performed at the T-13 vertebral level exposing the upper lumbosacral enlargement. The spinal cord was exposed using a 26G needle to open the dura. Injections of retrograde label (see below) were made using a 5 μl Hamilton syringe (Hamilton Company; Reno; NV) with a micropipette tip (30 μm inner diameter) glued to the syringe needle. The syringe was attached to a micrometer injection apparatus seated in a stereotaxic frame. A total of six 0.30 μl Fluorogold (FG; Biotinum Inc; Hayward; CA; 3% w/v in dH2O) or Dextran Tetramethyl Rhodamine 3,000 M.W. (DTMR; Molecular Probes; Eugene; OR; 1% w/v in 1 × PBS) injections were made bilaterally centered within the intermediate gray matter (laminae V - VIII) at the rostral, middle, and caudal aspects of the laminectomy site. The needle penetrated through the dorsal surface of the spinal cord for a distance of 1 mm on either side near the midline, and was then withdrawn 500 μm before beginning the injection. The injection was made over 5 minutes, leaving the needle in place for 1 minute prior to retraction to limit leakage of label.

#### Spinal Transection

one-week post-retrograde labeling, animals in experimental groups receiving a spinal transection were again anesthetized with ketamine/xylazine. A laminectomy was performed at the T9 vertebral level. Once the spinal cord was exposed a pair of iridectomy scissors (Fine Scientific Tools; Foster City; CA) was used to transect the cord, and with a successful transection the rostral and caudal edges of the cut spinal cord retracted exposing the underlying vertebrae. Following transection, a probe was scraped along the inner wall of the vertebral canal through the lesion site to further ensure a complete lesion. Gelfoam soaked in 0.9% isotonic saline was used to stop any bleeding.

#### Spinal Contusion

After appropriate tracer transport time (see Table 1 for details), animals were anesthetized with an IP injection of sodium pentobarbital (Nembutal 0.1 cc/100 g). Once areflexic, a laminectomy was performed at the T9/T10 vertebral level exposing the lower thoracic spinal cord. The animal was positioned and secured into the frame of an NYU Impactor by clamping the T8 and T11 spinous processes, followed by a moderate spinal contusion injury, dropping a 10 g rod from a height of 25 mm using the MASCIS protocol [18,19]. Successful injuries were determined by the Impactor analysis, visual inspection of the lesion under the surgical microscope, and complete acute loss of hindlimb responses after recovery from anesthesia.

#### Post-Operative Care

Following each surgical labeling or SCI procedure, animals were sutured using 3.0 silk to close the musculature, and 3.0 Nylon to close the skin; external sutures were removed after the first week post-operatively. All spinal injured animals received twice daily bladder expression until the micturition reflex returned, and injections of Cefazolin (Sandoz Inc; Princeton; NJ; 0.03 cc, SQ) were given twice daily for the first week following spinal injury to prevent urinary tract infections, or when infections occurred. Buprenorphine hydrochloride (Buprenex injectable; Ben Venue Laboratories Inc; Bedford; OH; 0.03 cc SQ) was given twice daily for the first 48 hrs post-operatively for pain management. All post-operative animals had *ad libitum *access to both food and water.

### Histology

#### FG Tissue

Following the assigned post-operative recovery time, animals were euthanized by an IP injection of sodium pentobarbital (Fatal Plus, 0.5 cc) and then decapitated. The mid thoracic spinal cord, T5-T8, was rapidly dissected, embedded in O.C.T compound (Tissue Tek^® ^embedding medium), and rapidly frozen on dry ice. Tissue samples were stored at -80°C until processing. Tissue sections were cut transversely at 16 μm using a cryostat and mounted on glass Superfrost Plus slides (Fisher; Pittsburgh; PA) or poly-ethylennaphtalae (PEN) foil slides (Leica; Wetzar; Germany) and maintained at -20°C during the sectioning process. Slides were stored at -80°C until laser microdissection (LMD).

#### DTMR Tissue

Animals were euthanized by an injection of sodium pentobarbital (Fatal Plus, 0.5 cc), and transcardially perfused with 500 ml 0.1 M 1 × PBS (pH 7.4) followed by 500 ml 4% paraformaldehyde (pH 7.4). Spinal cords were post-fixed in 4% paraformaldehyde for 24 hours followed by cryoprotection in 20% sucrose for a minimum of 24 hours. Tissue samples were embedded in O.C.T compound (Tissue Tek^® ^embedding medium) and frozen on dry ice. Tissue samples were stored at -20°C throughout sectioning and storage. Cryostat sections were cut in the transverse plane at a thickness of 20 μm, and slides containing tissue sections were stored at -20°C until further processing.

#### Immunohistochemistry

Retrogradely labelled DTMR-TPS neurons were probed immunohistochemically for various neurotrophic receptors (Trk A, B, C, GFRα1; 1:100, or Ret; 1:300; Santa-Cruz; Santa-Cruz; CA), a marker of cell stress (HSP-27 "Hsbp1"; Rabbit IgG; 1:200; Assay Designs|Stressgen; Ann Arbor; MI), a protein involved in axon regeneration (GAP-43;1:250; Millipore; Bellerica; MA), and a marker of cell death (activated caspase 3;1:250; Sigma-Aldrich; St. Louis; MO). All retrogradely labeled tissue was incubated in primary antibodies overnight at 4°C. Secondary labeling of GFRα1 and Ret was bioamplified, and visualized using strepavidin-FITC conjugate 1:300 in avidin buffer. Secondary antibody detection of all other primary antibodies used either a goat anti-rabbit or mouse - FITC conjugate (1:50; Zymed laboratories; San Francisco; CA) or goat anti-rabbit Marina Blue (1:300; Invitrogen; Carlsbad; CA). All sides were coverslipped with vectasheild (Vector Labs Inc; Burlingame; CA).

### Microarray Techniques

#### Laser Microdissection

**Figure 1:**
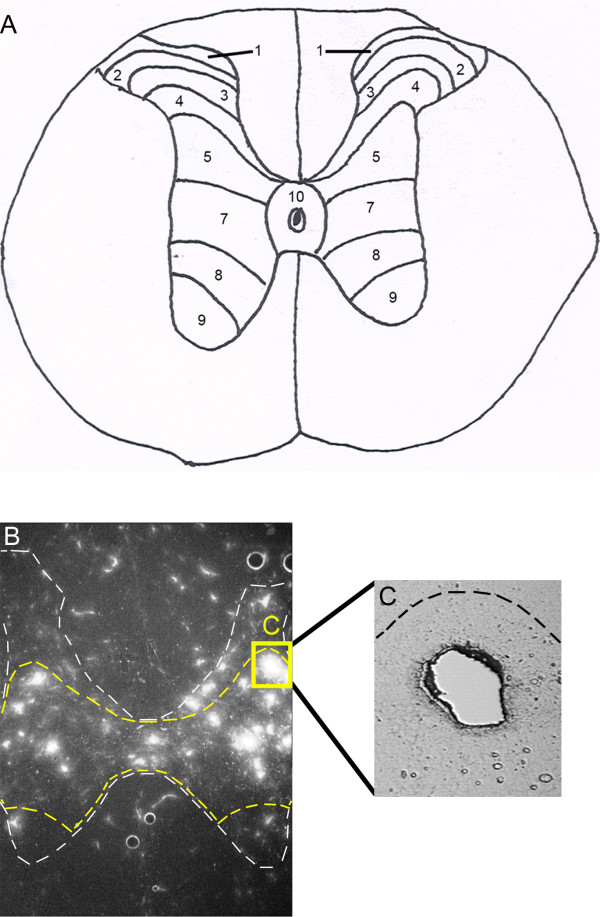
**Laser microdissection of thoracic propriospinal (TPS) neurons for microarray analysis**. ***A*, **TPS neurons selected for microdissection were located within laminae V, VII, VIII, and X of the thoracic spinal cord. ***B*, **Thoracic propriospinal (TPS) neurons retrogradely labeled by fluorogold (FG) injections into the lumbosacral enlargement are shown at 10 × magnification under an ultraviolet filter. The white dashed line marks the boundary of the white and gray matter, while the yellow dashed line outlines the laminae area from which neurons were collected. ***C*, **Region from which a FG labeled TPS had been individually laser microdissected yielding a relatively pure TPS neuron RNA message. Figure **1a **was adapted from: The Spinal Cord (Watson C, Paxinos G, Kayalioglu G ed.) p 270, New York, Academic Press

Tissue was mounted on poly-ethylennaphtalae (PEN) foil slides (Leica, Wetzar; Germany) that were maintained on dry ice until laser microdissection. Once a slide was removed from the dry ice, neurons were dissected over a period of 10 minutes. Slides were positioned on the stage of a Leica AS LMD microscope (Leica Microsystems; Bannockburn; IL). Retrogradely labeled FG-TPS cells were visualized under the UV filter using the 10 × objective (Figure 1B). Using the microscope's computer software, FG labeled cells located within the intermediate gray matter (laminae V, VII, VIII, and around the central canal, lamina X; Figure 1A,B) were individually dissected (Figure 1C); a minimum of 200 FG labeled neurons were collected from each animal.

#### RNA Purification & Amplification

Dissected neurons were collected directly into a PCR tube cap containing 30 μl RLT lysis buffer (Qiagen; Valencia; CA) with 1% β-marcaptoethanol (Sigma Aldrich; St. Louis; MO). RNA was then purified using the RNeasy Mini kit (Qiagen; Valencia; CA), eluted in 30 μl nuclease free water, and concentrated down to 10 μl by vacuum centrifugation. Total RNA concentration was determined by loading 1 μl of concentrated sample onto an RNA 6000 Pico Chip (Agilent Technologies; Santa Clara; CA). Acceptable RNA samples (those with only minimal or no evidence of degradation) were amplified and labeled using the Ovation™ Biotin RNA Amplification and Labeling System (NuGen; San Carlos; CA), producing a 10,000 fold amplification of the mRNA. Using the basic 3' Ribo-SPIA™ (NuGen; San Carlos; CA), the mRNA was then used to produce an enriched anti-sense cDNA probe for hybridization to the Rat Gene 1.0 ST Array (Affymetrix; Santa Clara; CA).

#### Microarray Hybridization

All microarray processing was performed using standard hybridization, washing, staining, and scanning protocols in the SUNY Microarray Core lab at Upstate Medical University. Following scanning, the raw array images were converted to .CEL file images for downstream analysis. All the array data for this study have been deposited at the NCBI Gene Ascension Omnibus (GSE20907, http://www.ncbi.nlm.nih.gov/geo/query/acc.cgi?acc=GSE20907)

#### RT-PCR

The reverse transcriptase reaction to convert the RNA probes into first strand cDNA for PCR was carried out using the RT^2 ^First Strand Kit (SA Biosciences; Frederick; MD) following the manufacturer's directions and using the supplied reagents for each RNA sample. PCR was performed using the RT^2 ^SYBR Green qPCR Master Mix and RT^2 ^Profiler™ PCR Array for Rat Neurotrophins and Receptors (SA Biosciences; Frederick; MD) following the manufacturers directions and using the supplied reagents. PCR plates were run on a Roche 480 LightCycler.

### Imaging and Data Analysis

#### Fluorescent Microscopy

All images were taken on a Zeiss Axio Imager A.1 microscope (Carl Zeiss, Germany). DTMR labeling was viewed under a CY3 filter, while the immunohistochemistry was viewed under either a FITC or DAPI filter. All images were captured using a SPOT RT slider camera, model 2.3.1 (Diagnostic Instruments; Sterling heights; MI). All digitized images where processed in the Spot™ Advanced software (v. 3.3.4 for Macintosh, Diagnostic Instruments Inc.), using the RGB color histogram tool to adjust both the image brightness and contrast. No other manipulations were made to these images.

#### Microarray

Quantile-normalized expression values were obtained for all genes using RMA normalization of the .CEL file data in GeneSpring GX (Agilent Technologies) and selected data formatted for import into Microarray Experiment Viewer 4.2 (MEV 4.2) [20]. Significant changes in expression of single genes were detected using a 2-way ANOVA to compare the effects of treatment across four time-points post surgery. The resulting P values from the main effect ANOVA were corrected for multiple testing using the Benjamini-Hochberg False Discovery Rate (FDR) algorithm.

Because the study design included assessments of expression changes at four discrete time-points post-SCI, we also chose to provide an overview of the genes with the most robust changes at any one or more particular time-points, whether or not that change generalized across all time-points. Thus, we compiled a table that contained lists of the genes with the largest significant increases in expression and the largest significant decreases in expression at one or more of the four time-points (see Additional File 1). From these lists, we selected the 20 genes with the largest increases and 20 genes with the largest decreases for hierarchical clustering and heat map visualization.

In addition to the hypothesis-free analysis of genome-wide expression data, we also determined whether there were significant expression changes in three specific custom-designed gene networks that we hypothesized would be critically involved in the response to injury. These networks included: (1) Regeneration Associated Genes and Cell-Survival and Neuroprotection Genes (RAG and CsNp Genes); (2) Surface Receptor and Growth Factor (GF) Genes; and (3) Apoptosis Genes. Complete lists of the genes in each of these networks are provided in the Additional Data section. Each network was compiled from only genes with expression values exceeding the lowest 15 percentile of all genes on the array. Within each network, significance was determined by permutation of the 2-factor (treatment × time) ANOVA matrix, with a P value cutoff of 0.01 based on 1000 permutations within MEV 4.2.

To determine the evidence for enrichment of significantly changed genes in specific biological pathways or gene networks, Ingenuity Pathway Assist (IPA) software (Ingenuity) was utilized. Data sets for each time-point were uploaded to IPA consisting of all the genes changed at the Benjamini-Hochberg FDR-corrected P value of 0.05 or less. These data sets were then filtered using IPA software to include only those genes which were annotated as expressed in any nervous tissue or immune cell as well as changed in expression by at least +/- 0.25 on a log2 scale. The top Bio Functions results were complied from each of the IPA analyses (see Additional Data section) for all gene networks containing 5 or more genes. Corrected significance values for each gene that mapped to these Bio Function networks were generated by IPA. Selected gene networks that showed up in multiple time-point analyses were used to generate biological interaction network/pathway diagrams in IPA for comparative purposes (see Additional Data).

#### qRT-PCR

The average Cp value for each gene of interest (GOI), both in non-injured controls and spinally transected animals, was calculated and then compared to a given set of reference genes included on the plate to give us the ΔCp value. For each GOI the ΔCp value of the spinally transected group value was subtracted from the mean ΔCp value of the control group to yield the ΔΔCp, from which the directional and fold change in our GOI were determined. PCR validation of our GOI was performed by plotting the log2 difference of the lesion versus control groups for both the PCR data and microarray data, and subsequently determining the Pearson correlation between the expression changes determined using these two approaches.

## Results

### Gene-Microarray analysis of the TPS intrinsic response to axotomy

#### Hypothesis -free single gene analysis

**Figure 2:**
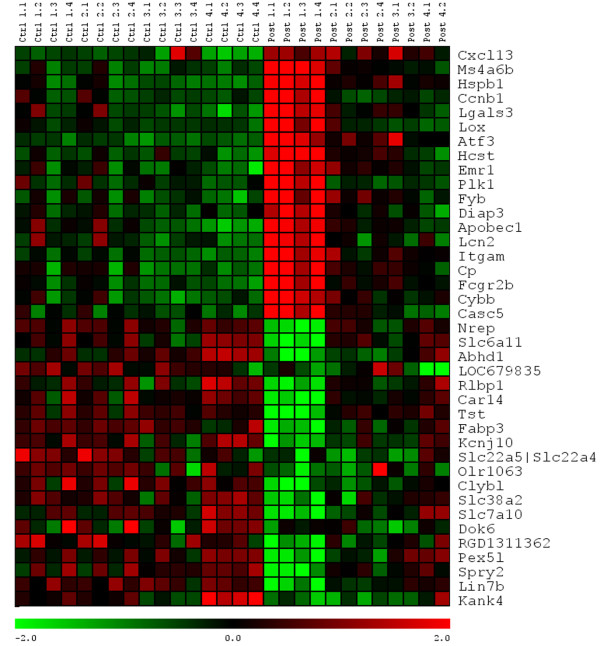
**Genetic expression profiles for the top 20 genes up-regulated and down-regulated following spinal transection**. The expression profiles illustrate the normalized log2 expression values with low expression values being represented by green and high expression in red. The 20 genes exhibiting the greatest up-or down- regulation were determined by using a 2-way ANOVA comparing the effects of treatment across post-operative survival points. The resulting P values from the main effect ANOVA were Benjamini-Hochberg corrected for multiple testing. Further investigation of the top 20-upregulated genes revealed a majority of them were either involved or associated with immune function and inflammation, or in cell maintenance and survival (Table 2.2). The top 20 genes down-regulated were mainly involved in different aspects of neuron cell biology. As illustrated in this expression profile, the maximal response (up- or down-regulation) is found during the first, three day, post-injury interval (Post 1.1-1.4). As time progresses, gene expression levels begin to wane (Post 2.1-2.4) returning to levels near to that seen in non-injured control animals by 1-month post-injury (Post 4.1-4.2). This overall analysis of the TPS response to a low thoracic injury suggests that the immune and inflammatory response plays a major role in the early post-injury response of TPS neurons.

We first initiated a genome wide, hypothesis free analysis to examine the overall response of TPS neurons to axotomy following a low thoracic spinal transection. The genes with significant main effects of SCI were used for subsequent gene network analyses. In addition, we compiled lists of the genes with the most robust increases or decreases in expression at one or more of the four time-points (see Additional File 1). Out of these lists, the expression profiles of the top 20 genes both up and down regulated are illustrated in Figure 2 following hierarchical clustering, and listed in Table 2.

**Table 2:** Top 20 Genes Up-regulated Following Injury

Probe ID	Gene Symbol	Gene Title	Max Log2 Δ	Known/Presumed Function *
10775731	Cxcl13	Chemokine (C-X-C motif) ligand 13	2.88	Immune
10728924	Ms4a6b	Membrane-spanning 4-domains, subfamily A, member 6B	2.57	Cell Signaling
10761128	Hspb1	Heat shock protein 27	2.50	Cell Survival
10821016	Ccnb1	Cyclin 1B	2.43	Cell Cycle
10779673	Lgals3	Lectin, galactose binding, soluble 3	2.33	Immune
10804463	Lox	Lysyl oxidase	2.32	ECM Modification
10770710	Atf3	Activating transcription factor 3	2.26	Transcription
10720565	Hcst	Hematopoietic cell signal transducer	2.22	Immune
10931222	Emr1	EGF-like module contaning, mucin-like, hormone receptor-like sequence 1	2.06	Cell-Cell Interaction
10710627	Plk1	Polo-like kinase 1	2.02	Cell Cycle
10813392	Fyb	FYN binding protein	2.00	Immune
10785479	Diaph3	Similat to Protein diaphanous homolog 3	1.99	Actin-Cytoskeletal
10865329	Apobec1	Apolipoprotein B mRNA editing enzyme, catalytic polypeptide 1	1.98	Transcription Modification
10844331	Lcn2	Lipocalin 2	1.97	Inflammatory
10711268	Itgam	Integrin alpha M	1.95	Immune
10814430	Cp	Ceruloplasmin	1.93	Development and Homeostasis
10769771	Fcgr2b	Fe fragment of IgG, low affinity Ilb, receptor	1.89	Immune
10936899	Cybb	Cytochrome b-245, beta polypeptide	1.85	Immune
10838733	Casc5	Similar to cancer susceptibility candidate 5	1.83	Kinetochore Formation
10858559	Clec4a3	C-type lectin domain family 4, member a3	1.82	Immune

Top 20 TPS neuron genes found to be up-regulated following a T9 complete transection. Examination of the genes found to be up-regulated revealed a majority of them to be involved with either the inflammatory or immune response. This list has the genes ranked according to their log2 change, and has been edited from the overall master list (Supplementary Data, Table S1) to exclude non-coding RNA sequences. (*) Known/presumed functions were obtained from the Crown Human Genome Center & Weizmann Institute of Science website GeneCards^® ^which can be accessed at http://www.genecards.org/index.shtml

The pattern of gene expression (Figure 2) illustrates that the most significant change occurs 3-days post injury (p.i.; Post 1.1-1.4), and begins to wane by 1-week p.i. (Post 2.1 - 2.4). At the 1-month time-point (Post 4.1 and 4.2) the expression levels for most genes appear to return near the level of uninjured control animals. Initially, genes exhibiting a significant down-regulation appear to be mainly involved with different aspects of neuronal cell biology (Table 3), with no one aspect of cell biology being heavily represented. The genes exhibiting the strongest up-regulation are primarily involved with the immune/inflammatory response (Cxcl13, Ccnb1, Hcst, Fyb, Lcn2, Itgam, Fgcr2b, Cybb, and Clec4a3; Table 2), or cell maintenance/survival/stress (Ms4a6b, Hspb1, Ccnb1, Atf3, Emr1, Plk1, Diap3, Cp, and Casc5; Table 2).

**Table 3:** Top 20 Genes Down-Regulated Following Injury

Probe ID	Gene Symbol	Gene Title	Max Log2 Δ	Known/Presumed Function *
10803692	Nrep	Neuronal regeneration related protein	-1.70	Nerve Outgrowth
10857916	Slc6a11	Solute carrier family 6 (neurtransmitter transporter GABA), member 11	-1.40	Neurotransmitter Uptake
10888931	Abhd1	Abhydrolase domain containing 1	-1.19	Multiple Enzymatic
10862194	LOC679835	Similar to Anionic trypsin II precursor gene	-1.18	**Uncharacterized**
10722918	Rlbp1	Retinaldehyde binding protein 1	-1.14	Visual Cycle
10825100	Car14	Carbonic Anhydrase 14	-1.12	Homeostasis
10905284	Tst	Thiosulfate sulfurtransferase	-1.00	Fe-S Complex Formation
10872473	Fabp3	Fatty acid binding protein 3, muscle and heart	-0.89	Fatty Acid Transport
10765740	Kcnj10	Potassium inwardly-rectifying channel, subfamily J, member 10	-0.86	Voltage Gates
10742645	Slc22a5	Solute carrier family 22 (organic cation/carnitine transporter), member 5	-0.83	Carnitine Transporter
10893412	Olr1063	Olfactory receptor 1063	-0.83	**Uncharacterized**
10782144	Clybl	Citrate lyase beta like	-0.82	Citrate Lyase Activity
10906608	Slc38a2	Solute carrier family 38, member 2	-0.81	Amino Acid Transporter
10706134	Slc7a10	Solute carrier family 7 (neutral amino acid transporter, y+ system), member	-0.80	Glutamatergic Neurotransmitter Modulation
10805605	Dok6	Docking protein 6	-0.80	Neurite Outgrowth
10896666	RGD1311362	Similar to hypothetical protein FLJ10204	-0.79	**Uncharacterized**
10822735	Pex51	Peroxisomal biogenesis factor 5-like	-0.78	Peroxisomal Targeting
10785773	Spry2	sprouty homolog 2	-0.77	FGF Antagonist
10721796	Lin7b	Lin-7 homolog b	-0.72	Channel and Receptor Localization
10878210	Kank4	KN motif and ankyrin repeated domains 4	-0.72	Actin Stress Fiber Formation

Top 20 down-regulated genes encompass broad functions of cell biology with no one specific function being heavily represented. This list has the genes ranked according to their log2 change, and has been edited from the overall master list (Supplementary Data, Table S1) to exclude non-coding RNA sequences. (*) Known/presumed functions were obtained from the Crown Human Genome Center & Weizmann Institute of Science website GeneCards^® ^which can be accessed at http://www.genecards.org/index.shtml

#### Hypothesis-based gene network analyses

We also determined which biological pathways or gene networks are most consistently affected in TPS neurons following spinal transection using Ingenuity Pathway Assist (IPA) software (Ingenuity) to map those genes with significant main effects across all time-points. Three days p.i. 39 biological pathways/gene networks containing 838 genes are significantly affected with the top 6 being: antigen presentation (52 genes), inflammatory response (52 genes), immune cell tracking (52 genes), hematological system development and function (52 genes), cell to cell signaling and interaction (52 genes), and cell-mediated immune response (44 genes) (See Additional File 2) the same gene can be involved in more than one related pathway/network). By one week p.i, there is a much more focused response with only 3 pathways/gene networks significantly affected containing many fewer genes (32 genes) than at 3-days p.i.. There is also a large shift from immune related genes to genes involved in nervous system development (18 genes) cellular growth and proliferation (9 genes), and neurological disease (5 genes). Only 5 of 27 significantly changed genes in the nervous system development and function category at 3-days p.i. are among those changed 1-week p.i.. Similarly only 1 of the 16 genes changed in the cellular growth and proliferation category at 3-days p.i. and 1 of 30 genes changed in the neurological disease category are among the genes changed at 1-week p.i. in these categories. The gene in these later two categories is Casp3.

By two weeks p.i. there was a significantly expanded response with 12 gene networks comprised of 70 genes being affected according to the IPA analysis. The top 3 pathways/networks involved are hematological system development and function (14 genes), cellular movement (7 genes), and inflammatory response (7 genes). Only 5 of the 52 genes changed in the hematological development and function at 3-days p.i. are among the 14 genes significantly changed in this category at 2-weeks. Similarly only 2 of 32 genes significantly changed at 3-days p.i. in the cellular movement category are among the 7 genes changed at 2-weeks p.i.. Only 1 of the 52 genes significantly changed in the inflammatory response category at 3-days p.i. is among the 7 genes changed in this category at 2-weeks p.i..

At 1 month p.i., 234 genes involved in 34 different gene pathways/networks were significantly changed. The top three networks are nervous system development and function (14 genes), neurological disease (10 genes), and cell death (9 genes). Again, few of the 14 genes significantly changed in the nervous system development and function category at 1-month p.i. are also changed at 3-days (4 of 27), at 1-week (3 of 12), or at 2-weeks p.i. (1 of 6), and only Atf3 is changed at all the time-points other than 1-week p.i.. Many of the significantly changed genes in the other two categories at 2-weeks p.i. are found in both categories at this survival time since they overlap in function. However few of these genes were significantly changed at earlier survival times. 17 genes are also significantly changed at 1-month p.i. in the hematological development and function category compared to control expression level. Again, only 1 of 52 genes in this category at 3-days p.i. is also changed at 1-month p.i.

### IPA Biological Pathway/Network Analysis

**Figure 3:**
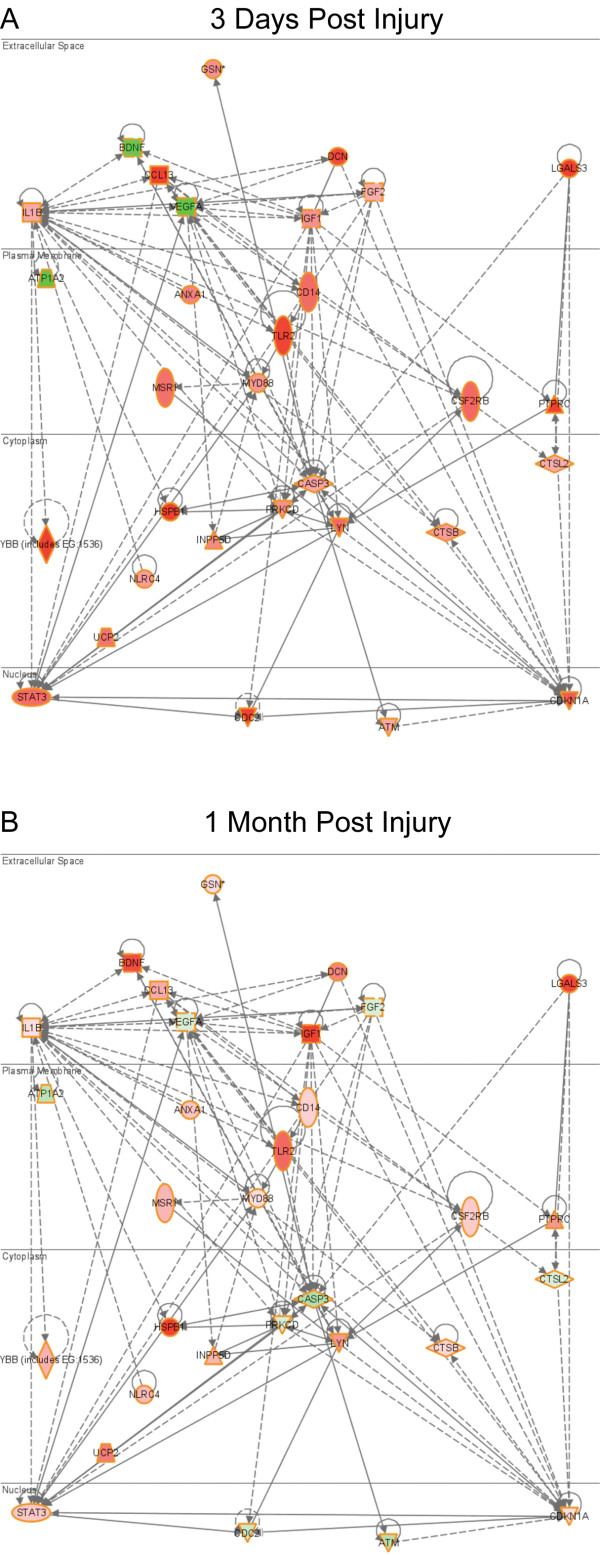
**Ingenuity Pathway Assist (IPA) analysis of significant genes involved in cell death**. Data sets for each time point were uploaded to IPA that consisted of all the genes changed at the Benjamini-Hochberg FDR-corrected P value of 0.05 or less. These data sets were then filtered using IPA software (See Materials and Methods). IPA biological/gene network analysis indicates the interaction of groups of genes with one another and how their expression decreases (in green) or increases (in red) over time. In addition to the visualization of the gene interactions, IPA analysis shows the sub-cellular localization of each of the molecules. See text for details. For conventions and symbols, refer to Ingenuity website legend description https://analysis.ingenuity.com/pa/info/help/help.htm#legend.htm.

Gene networks expressed commonly at all four post-injury time-points (inflammatory response, cell death, nervous system development and function, neurological disease, and cell growth and proliferation) were further explored by composing functional biological network/pathway analysis using IPA software (See Additional Files, 3, 4, 5, 6, 7 respectively). These pathways allowed us to observe how each gene in a pathway interacts with one another, and how, over time, the expression of these genes and the sub-cellular localization of each of the molecules changes. For example, the specific gene network illustrated in Figure 3 shows how genes regulating cell death interact with each other, and how their expression (using the same color scale as the expression profile) changes from 3-days p.i. (Figure 3A) to 1-month p.i. (Figure 3B; for complete time-point comparison see Additional File 4). This specific gene network illustrates that 3-days following injury, brain derived neurotrophic factor (BDNF) is down-regulated, while the pro-apoptotic gene caspase3 (Casp3) is upregulated. These two genes exhibit an inverse relationship with BDNF increasing in expression and Casp3 decreasing in expression 1-month p.i.. This pathway also illustrates the interactions of Casp3 with protein kinase C delta, (Prkcd), which has been demonstrated to be involved with apoptosis, and with ataxia telangiectasia mutated (Atm), thought to be activated upon signaling of apoptotic stresses.

### Custom-designed gene network analysis

In addition to an undirected (i.e. hypothesis free) evaluation of the top changed genes and biological pathways, three custom-designed gene lists were generated to test whether these genes were specifically affected by axotomy/spinal injury; regeneration associated/cell stress and neuroprotection genes, growth factor genes, and apoptosis-related genes (see below). Once the gene lists were run through a 2-way ANOVA (see below) we observed a significant change in expression in only 29 out of a total 679 genes in three groups of interest (regeneration/survival, growth factors/receptors and apoptosis), the greatest percentage were in the RAGs/CsNp gene list. We determined if there was statistical evidence for over-representation of significantly changed genes in these groups based on a hypergeometric P value (equivalent to a 1-sided Fischer's exact test). For the entire set of 24,008 permuted genes examined, only 969 were changed at the .01 alpha level using our 2 way ANOVA (4.0% of the total genes) as described in the Materials and Methods. This compares to 14 significantly changed RAG/CsNp genes out of 84 tested (16.7%), 8 significantly changed growth factor genes out of 214 tested (3.7%), and 7 significantly changed apoptosis genes out of 381 tested (1.8%). This hypergeometric analysis indicates that the 4.1 fold greater enrichment in the RAG/CsNp genes was highly significant (p = 0.000015) whereas there was no overall change in growth factor receptor genes (-1.1 fold, p = .42) and significantly fewer genes were found in the apoptosis gene group than expected by chance alone (-2.2 fold, p = .02). The under-representation of apoptosis genes indicates that only a small number of these genes is affected by spinal injury, all of which were pro-apoptosis (see below). In fact, this finding was also indicated by the IPA analysis (see Figure 3, Additional File 4).

### Thoracic propriospinal neurons upregulate both regeneration associated and neuroprotective survival genes following a low thoracic axotomy

Our list of regeneration associated genes (RAGs) and cell survival and neuroprotection (CsNp) genes was compiled using the query search available on the Affymetrix NetAffx website http://affymetrix.com as well as genes selected from the axonal regeneration and cell survival/neuroprotection literature. We analyzed the response of 84 genes of interest (GOI; see Additional File 8). GOI that exhibited significant changes in expression were selected using a two-factor ANOVA analysis (comparing the effect of treatment, spinal transection vs. control, and post-injury survival time) with significance set at the *p *≤ *0.01 *level based on 1000 permutations.

**Figure 4:**
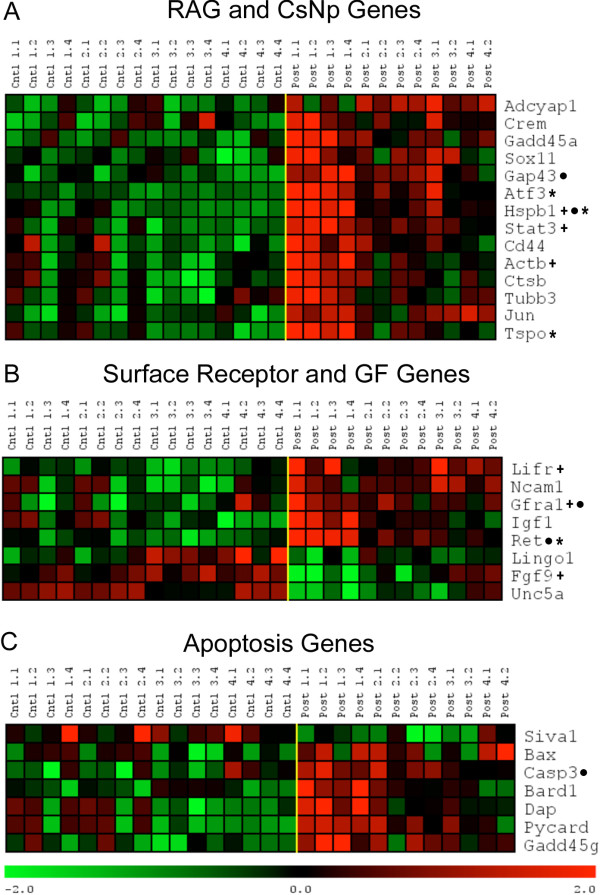
**Expression profiles of specific genes classes significantly altered following spinal transection**. The expression profiles illustrate the normalized log2 expression values with low expression values being represented by green and high expression in red. The three expression profiles illustrated here were generated using a combination 2-factor ANVOA with a significance of (*p = 0.01*, based on 1000 permutations) and cluster analysis to reveal the expression patterns. ***A*, **Expression profiles for 14 of the 84 genes associated with regeneration (RAG) or cell survival and neuroprotection (CsNp) using the ANOVA analysis demonstrated a significant change in expression following injury. As shown, all were upregulated. ***B*, **The expression profile for genes associated with surface receptors, neurotrophic receptors, and neurotrophins and growth factors (GF). ANOVA analysis revealed 8 of the 214 genes examined exhibited a significant change in expression following the injury, 3 of which (Unc5a, Lingo1, Fgf9) are down-regulated. ***C*, **Even though both pro- and anti-apoptotic genes were included, all seven genes revealed by this analysis are pro-apoptotic. Only one of the seven genes is down regulated (Siva1), while the other 6 demonstrate a sharp up-regulation following axotomy. ***A, B, C ***All three-expression profiles have a common pattern in gene expression that matches the pattern found for the overall significant response (see Figure 2). Asterisk indicates genes from the genome wide hypothesis free analysis also identified in our specific gene programs. Plus sign indicates genes whose expression values were validated by qRT-PCR. Black circle indicates genes validated immunofluorescently.

Fourteen of the 84 GOI exhibited a significant change in expression for at least one out of the four time-point comparisons (3-days, 1-week, 2-weeks, and 1-month p.i.). Eleven of the 14 genes (Table 4; Adycap1, Crem, Sox11, Gap43, Atf3, Stat3, Cd44, Actb, Ctsb, Tubb3, and Jun) were involved with the process of regeneration, while the other 3 genes (Gadd45a, Hspb1, and Tspo) were mainly involved with cell survival and neuroprotection, although Hspb1 also has been associated with axonal regeneration [21]. All 14 genes exhibited a strong up-regulation in expression 3-days post injury (Figure 4A; Post 1.1 - 1.4) that was diminished by 1-week post injury (Post 2.1 - 2.4). Furthermore when the change in gene expression 1-week p.i. was compared to the expression level of the appropriate control, no significant (*p < 0.01*) change was detected. However, despite the lack of statistical significance in the changes of gene expression, the array data reveal that the levels of expression in the injured neurons remain elevated above those observed in controls throughout the later p.i. periods for many of the RAG and CsNp genes.

**Table 4:** Specific Regeneration Associated and Neuroprotective Genes of Interest Exhibiting Significant Change in Expression Post-Injury

Gene Name	Symbol(s)	Reference
Actin, beta	Actb	[76]
Adenylate cyclase activating polypeptide 1	Adcyap1, Pacap	[77,78]
Activating transcription factor 3	Atf3	[8,79-82]
CD44 Molecule	Cd44	[23]
cAMP response element modulation	Crem, Icer	[83]
Cathepsin B	Ctsb	[84]
Growth arrest and DNA-damage-inducible 45 alpha	Gadd45a	[85]
Growth-associated protein 43	Gap43	[8,86-88]
Heat shock 27 kDa protein	Hspb1	[79,82,89-91]
SRY-related HMG-box gene 11	Jun	[8,23,92,93]
Jun oncogene	Sox11	[83,94]
Signal transducer and activator of transcription 3	Stat3	[23,93]
Translocator protein (18 kDa)	Tspo, Bzrp	[95,96]
Tubulin, beta 3	Tubb3, Tuj1	[97]

Specific genes from our complied lists of genes of interest (GOI) that demonstrated a significant (*p = 0.01*) change in expression following T9 axotomy, as determined by a 2-factor ANOVA. ***3.1*, **Genes known to be associated with axonal regeneration, cell survival and neuroprotection. These gene lists are arraigned in alphabetical order and include both the symbol and full gene name. Also included in this table are a series of references used to classify these genes into their specific category.

### Thoracic Propriospinal Neurons up-regulate several neurotrophic receptors and down-regulate several receptors inhibitory to axonal growth

The GOI (gene of interest) list that was complied for surface receptors and neurotrophic agents used the query search available on the Affymetrix NetAffx website. This GOI list also contained genes for growth factors and extracellular matrix (ECM) molecules. The final compiled GOI list contained a total of 214 genes (see Additional File 9) and was analyzed using the 2-factor ANOVA to determine which genes demonstrated a significant change in expression. Eight of the 214 genes demonstrated a significant change in expression following injury (Table 5). Five of the 8 genes were up-regulated (Figure 4B); 4 are surface receptor genes (Lifr, Ncam1, Gfra1, Ret) that can bind potent neurotrophic or growth promoting ligands i.e., glial cell-line derived neurotrophic factor (GDNF) or leukemia inhibitory factor (LIF). GDNF and LIF have been shown to promote neuron survival and/or axonal sprouting/regeneration [22,23]. The fifth gene is for a potent growth factor (Igf1) associated with axonal regeneration [2,23,24]. Three genes exhibited a significant down-regulation following axotomy: Fgf9, which is commonly associated with nervous system development [25]; Unc5a, involved with mediating repulsive axonal guidance cues; and Lingo1, which negatively regulates the process of myelination [26-28]. Lingo1 is also a member of the surface receptor complex that mediates the inhibitory cues mediated by myelin proteins, hindering the process of axonal regeneration [29]. The surface receptor expression profiles (Figure 4B) for these genes also exhibit a pattern of expression similar to what was observed in the RAG and CsNp genes (Figure 3A). All 8 genes showed a strong change in expression 3-days p.i. (Post 1.1-1.4) which started to wane 1-week p.i. (Post 2.1-2.4), and eventually approached the expression levels found in controls by one month p.i. (Post 4.1-4.2).

**Table 5:** Specific Surface Receptor and Neurotrophic Factor Genes of Interest Exhibiting Significant Change in Expression Post-Injury

Gene Name	Symbol(s)	Reference
Fibroblast growth factor 9	Fgf9	[25]
GDNF family receptor alpha 1	Gfra1	[22,97-99]
Insulin-like growth factor 1	Igf1	[2,23,24]
Leukemia inhibitory factor receptor	Lifr	[23]
Leucine rich repeat and Ig domain containing 1	Lingo1	[26,27,29,53,100,101]
Neural cell adhesion molecule 1	Ncam	[22,23,99,102]
Ret proto-oncogene	Ret	[22,97,98]
Unc-5 homolog A	Unc5a	[28,55,103]

Specific genes from our complied lists of genes of interest (GOI) that demonstrated a significant (*p = 0.01*) change in expression following T9 axotomy, as determined by a 2-factor ANOVA. Genes specific to surface receptors, growth factor, and neurotrophic agents. These gene lists are arraigned in alphabetical order and include both the symbol and full gene name. Also included in this table are a series of references used to classify these genes into their specific category.

### Thoracic Propriospinal Neurons upregulate many pro-apoptotic genes following low thoracic axotomy

Using the Affymetrix website, a query search for pro-, anti-, and apoptosis-related genes was conducted resulting in a compiled list of 381 genes (see Additional File 10). Following analysis using 2-factor ANOVA, only 7 genes (Table 6) of the 381 complied exhibited a significant (*p *≤ *0.01*) change in expression following transection injury. However, 6 of the 7 genes were up-regulated, while only 1 gene was down-regulated following axotomy (Figure 4C). The genes up-regulated following injury are all pro-apoptotic (See Table 6 for gene references). These genes either inhibit cell growth inducing apoptosis, Gadd 45γ; activate the caspase cascade resulting in apoptosis, Casp3; accelerate cellular apoptosis by antagonizing the Bcl2 apoptotic repressor, Bax; promote apoptosis via activation of caspase 9, Pycard; or have been documented to be pro-apoptotic or indicative of apoptosis, Bard1 and Dap. The one down-regulated gene, also pro-apoptotic, was apoptosis-inducing factor, Siva1.

**Table 6:** Apoptotic genes of interest exhibiting significant change in expression post-injury

Gene Name	Symbol(s)	Reference
BRCA1 associated RING domain 1	Bard1	[104]
BCL2-associated × protein	Bax	[105,106]
Caspase 3	Casp3	[23,105,107,108]
Death-associated protein	Dap	[109,110]
Growth arrest and DNA-damage-inducible 45 gamma	Gadd45g	[111]
PYD and CARD domain-containing protein	Pycard, Asc	[112,113]
SIVA1, apoptosis-inducing factor	Siva1	[114]

Specific genes from our complied lists of genes of interest (GOI) that demonstrated a significant (*p = 0.01*) change in expression following T9 axotomy, as determined by a 2-factor ANOVA. Genes associated with the process of apoptosis. These gene lists are arraigned in alphabetical order and include both the symbol and full gene name. Also included in this table are a series of references used to classify these genes into their specific category.

The expression pattern of the apoptotic genes again followed the overall pattern for the other specific GOI described above: i.e. expression was highly up- or down-regulated 3-days post axotomy, but diminished 1-week post axotomy returning to levels similar to those found in the control animals, in most cases by the second week post-SCI. The IPA analysis of the cell death network described above also shows some of the details of the apoptotic response that were described earlier (see Figure 3, Additional Files 2 and 4).

### Validation of the gene expression array using qRT-PCR

For validation of the microarray data, we selected the SA Biosciences Rat Neurotrophin PCR Array (see Materials and Methods). This array was chosen because it contained pre-validated primer sets for three reference genes (Rplp1, Rpl13a, and Ldha) that were unchanged in our array data, six genes that showed a Benjamini-Hochberg corrected significant effect of treatment (Actb, Hspb1, Gfra1, Fgf9, Lifr, and Stat3), as well as several other neurotrophin-related genes that showed a nominally significant effect of treatment (e.g., Gfra2, Ngfrap1, and Npy) that did not survive FDR correction (see Methods section). The PCR array results confirmed the significant changes in expression seen for the six genes with significant effects of treatment in the microarray analysis, as well as for three genes with nominally significant treatment effects.

**Figure 5:**
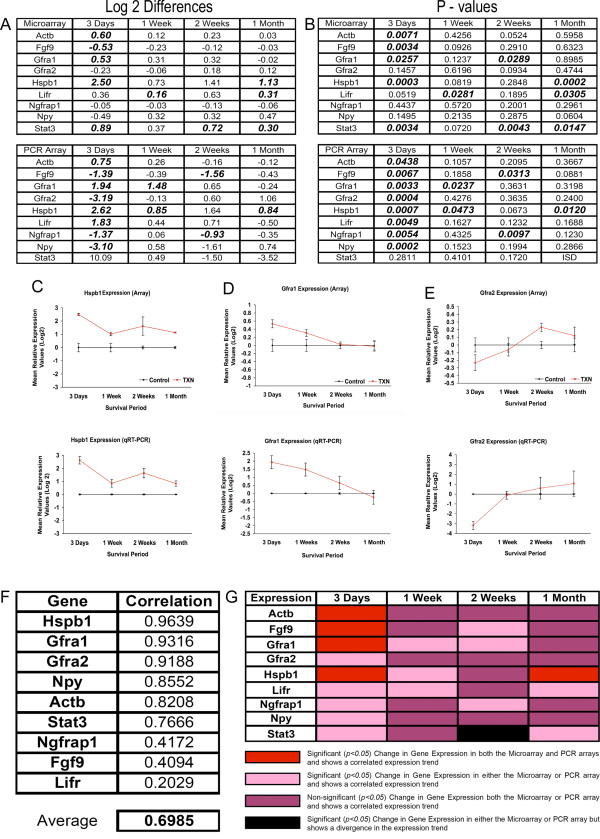
**PCR validation of gene expression data**. ***A*, **The log2 difference was obtained to give the direction of change in the expression values. ***B*, **P values for the significance in change of expression were determined using a two-tailed student t-test, for each time point comparison. ***A, B*, **Values appearing both bolded and italicized are genes demonstrating a significant change in expression (P-value). ***F***, A correlation analysis between the log2 differences of expression in gene array and PCR array was run to see if the expression trends observed in the gene array data corroborated the expression trends displayed in the PCR array. The overall correlation of 0.69 reveals a reasonable. ***C, D, E*, **The top three correlated genes (Hspb1, Gfra1, Gfra2) are plotted to illustrate the correlated expression trends between the log2 differences of the gene array data and PCR data. ***G*, **Summary of the data presented in panels A, B and F. Genes demonstrating a significant and correlated change in expression following injury in both the gene and PCR arrays are indicated in red (Actb, Fgf9, Gfra1, Hspb1). Genes with a significant change in expression in either the gene array or PCR array and demonstrate correlated expression trends are indicated in pink. Time points where no significant change in expression was found, but a correlation of expression between the two arrays is found are indicated in purple, The one time point where a significance in change of expression is found but the expression trends diverge is indicated in black.

For each of the nine genes listed above we next sought to make direct comparisons at each time-point between the microarray and PCR array findings. This was accomplished by compiling the log2 differences in expression that were determined by both techniques, calculating a Pearson correlation between these values, and testing for significant treatment effects at each time-point in both data sets with a student's t test (Figure 5). In general, we observed relatively strong correlation between the changes in gene expression observed with both techniques (Figure 5F). When the microarray and PCR array data for the top three correlated genes were plotted (Figure 5C-E), the observed expression patterns were highly similar confirming the results of the correlation analysis (Figure 5F).

As summarized in Figure 5G, the microarray and PCR array data corroborate each other. The 9 genes examined showed a significant change in expression 3-days post axotomy. Four of these genes (Actb, Fgf9, Gfra1, Hspb1) demonstrated both a significant change in expression and a correlated expression trend in both arrays, while the remaining 5 genes (Gfra2, Ngfrap1, Npy, Lifr, Stat3) showed a significant change in expression in either the PCR array or microarray but still demonstrated a correlated mean relative expression trend. At one week post-axotomy, many of the significant changes in expression were lost according to PCR array analysis, with only Hspb1 and Gfra2 still showing a significant change in expression, similar to what was observed in the microarray data.

Only at one point in our validation comparisons did we find a discrepancy in the PCR and microarray data, and that was the expression of Stat3 at 2-weeks post axotomy. This discrepancy could have been due to the fact that the PCR conditions for Stat3 were not optimal (only half of the reactions yielded Cp values) and/or the expression level of Stat3 was so low that it was not detected in some of our samples. It is also possible that alternative splicing may have occurred, which could have yielded different results because the array data interrogated the entire mRNA, while real time qPCR only amplifies a very small segment (usually one exon).

### Immunofluorescent validation of the TPS neuronal response to injury

**Figure 6:**
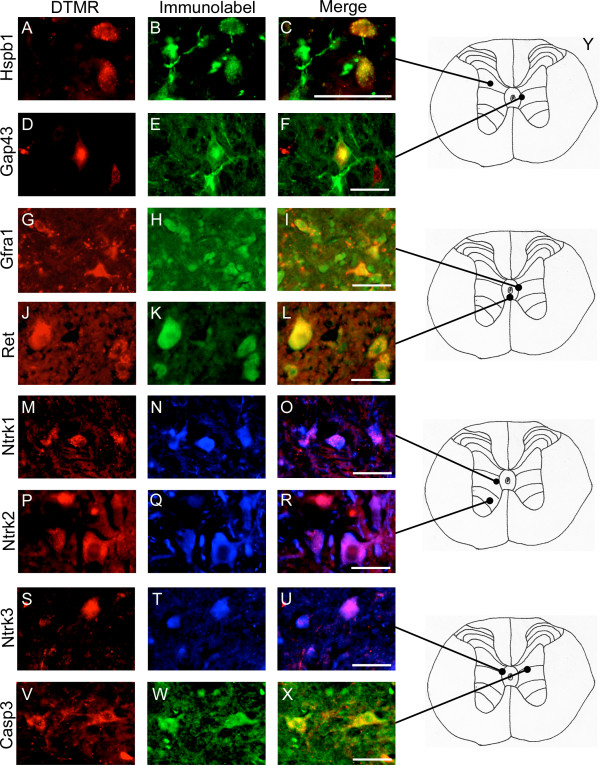
**Immunofluorescent analysis of the TPS neuronal response to a low thoracic contusion injury 1-week post-injury**. ***A, D, G, J, M, P, S, V*, **TPS neurons were retrogradely labeled by bilateral injections into the intermediate gray matter of the upper lumbosacral enlargement with DTMR 4 days prior to the injury, and 1-week following injury in cryostat sections (Cy3 Filter). ***B, E, H, K, N, Q, T, W*, **These sections also were immunolabeled by 1 of 8 antibodies. Examples show immuno-labeling within thoracic neurons (FITC Filter) related to cell survival, Hspb1; regeneration, Gap43; growth factor receptors, Gfra1, Ret, Ntrk1, Ntrk2, Ntrk3; and apoptosis, Casp3. ***C, F, I, L, O, R, U, X*, **TPS neurons colocalizing with the immuno-label for all 8 proteins are shown in these merged images. ***Y*, **Cartoons show the region of the intermediate gray matter from which the digital images were taken. All images taken at 40x; Scale bars = 100 μm.

Our previous work indicates that most TPS axons are severely damaged by low thoracic (T9) spinal contusion injury [30]. To confirm that the observed changes in gene expression in the transection model are an accurate portrayal of the TPS neuronal response to contusion injury, the expression of 8 different proteins (Gap43, Hspb1, Gfra1, Ret, Casp3, Ntrk1, Ntrk2, and Ntrk3) were examined immunofluorescently in DTMR retrogradely labeled TPS neurons following a moderate spinal contusion injury (NYU Impactor; 25 mm, 10 g weight drop). Comparisons were made at the 1-week post-injury time point in order to minimize confounding results due to lesion site debris-induced autofluorescence apparent at 3 days p.i.. Immunofluorescent analysis of the response of TPS neurons to a T9 contusion injury (Figure 6), mirrors the expression pattern revealed by gene array one week post-transection. Hspb1 immuno-labeling was evident in the majority of DTMR labelled cells 1-week post-contusion (Figure 6A-C). Gap43 immunoreactivity was only apparent in a small percentage of the DTMR labeled neurons 1-week p.i. (Figure 6F), being colocalized in one of the two DTMR labeled TPS neurons shown. However, axonal immuno-labeling with Gap43 was apparent throughout the intermediate gray matter. Activated Caspase3 immuno-labeling (Figure 6V-X) was also found in a proportion of the DTMR labeled TPS neurons (Figure 6X) at this time-point. There was extensive double labeling of DTMR retrogradely labeled TPS neurons 1-week post-contusion with either Gfra1 or Ret (Figure 6I,L). The majority of DTMR labeled TPS neurons were also immuno-labeled by Gfra1 or Ret in uninjured control sections indicating that these receptors are normally present in TPS neurons. Because our tissue sections were not stringently controlled to allow a quantitative analysis, we were unable to determine if the amount of immuno-labeling was increased post-contusion.

No significant change in the expression of the neurotrophic factor receptors Ntk1 (Trk A), Ntk2 (Trk B) or Ntrk3 (Trk C) was seen in the microarray or PCR array analyses. However, these three Trk receptors were included in our immuno-labeling analysis because of the key role they play in mediating the effect of neurotrophic factors (NGF, BDNF, and NT-3, respectively) on cell survival and axonal growth [4,31,32]. Immuno-labeling for Ntrk1 (Trk A) Ntrk2 (Trk B), and Ntrk3 (Trk C) (Figure 5N,Q,T) colocalized (Figure 6O,R,U) in many DTMR labeled TPS neurons (Figure 5M,P,S). However when the post-axotomy staining was compared to non-injured controls (data not shown) no noticeable difference in the amount of immuno-labeling within individual TPS neurons or the relative number of TPS neurons that were immuno-labeled by each neurotrophin receptor antibody was observed (data not shown).

## Discussion

Microarray technology has been used previously to examine the early genetic response following SCI [15,24,33-36]. However, most investigations studied the lesion site and/or nearby damaged spinal cord in its entirety and focused mainly on the acute or sub-acute period, ranging from a few hours to several days, and did not follow changes in gene expression over time [37]. Studies examining genetic changes of long tract neurons post-SCI, such as the corticospinal tract, usually have used *in situ hybridization*. This method is able to resolve genetic changes at a cellular level but is able to analyze only a small number of selected genes [7,38].

The present study expands on previous studies in several important ways. First, we used LMD to specifically collect individual FG retrogradely prelabeled TPS neurons at different times after axotomy. Additionally, our use of the Affymetrix Rat 1.0 Gene ST Array allowed us to examine the post-injury changes that occurred within the entire *Rattus norvegicus *genome. Finally, rather than focusing on the acute injury response during the first 24 hours after SCI, our analysis began 3-days post injury (p.i.), and continued until 1-month p.i., a longer period than analyzed in most previous studies.

One potential criticism of using FG to prelabel TPS neurons is that FG may have cytotoxic effects on retrogradely labeled neurons over time [39,40]. Therefore, we used separate control groups with similar post-FG injection survival times to compare at each interval examined post-SCI. Little difference was found comparing these control groups with each other in our microarray analysis and no evidence of a pro-apoptotic response was detected, further eliminating this potential variable from confounding our findings.

The microarray gene expression data for 14 genes of interest was validated using a combination of qRT-PCR and/or immunohistochemistry (IHC). While the spinal transection model was utilized for the microarray expression data to ensure all harvested FG labeled neurons were axotomized, we used a spinal contusion injury model for IHC validation. This second model also axotomizes most TPS neurons [30], but results in greater secondary injury processes and continued injury expansion p.i. than spinal transection [41,42]. Additionally, spinal contusion injury accounts for most spinal cord injuries in human beings (SCI network).

One limitation of the present study is that because of the complex experimental design, large numbers of different groups compared, and small numbers of samples per group (N = 4), the FDR correction we applied to identify genes exhibiting significant main effects of the SCI in the hypothesis-free genome-wide analysis is likely to be overly stringent, since many genes clearly showed changes at only one or two of the time points examined. Evidence of this was obtained by the qRT-PCR validation, which revealed three additional genes (Gfra2, Ngfrap1, and Npy) exhibiting significant changes in gene expression that only achieved nominal significance in the microarray analysis. To circumvent this problem, we specifically designed three gene lists that we hypothesized would be critically involved in the response to injury (RAG and CsNp genes, GF genes, and Apoptosis Genes) determining significance at each time point by performing a 2-factor ANOVA, (treatment × time), with a P value cutoff of 0.01 based on 1000 permutations. The hypergeometric analysis of these data indicated a highly significant 4.1 fold enrichment of RAG/CsNp genes suggesting an integrated gene network of genes involved in regeneration and neuroprotection. Such a network effect was not found for the other two gene lists analyzed, but a small group of upregulated pro-apoptotic genes, and growth factor receptor genes was revealed by this analysis. Additionally, we used IPA analysis for evidence of enrichment of significantly changed genes in specific biological pathways/gene networks. To gain a broader view of the genetic response, the data uploaded to IPA consisted of all the genes significantly changed at a P value of 0.05 or less after FDR correction, and further filtered so only genes expressed in the nervous or immune systems would be analyzed (see Materials and Methods for detailed information).

### TPS neurons mount an early but transient regenerative response post-axotomy

One factor previously shown to be important in the cell body response to axotomy is the distance between the location of the axotomy and the neuron's cell body. In this study, the neurons analyzed were collected from the T6-T7 spinal segment, two segments rostral (approximately 2 mm) from the lesion site. Besides the axotomy being close to the cell body, TPS neurons are located near the lesion site with the associated inflammatory and immune responses that occur in this region. Both of these factors are likely to play a role in the regenerative response of TPS neurons (see below).

Our microarray expression data and qRT-PCR experiments confirm that TPS neurons mount a robust regenerative response 3-days p.i. Genes demonstrating a significant up-regulation following injury at the *p *< 0.01 level include a group of genes classically associated with axonal regeneration (RAGs) including: Jun, Atf-3, Hspb1 (HSP-27), Adcyap1, Gadd45a, Gap43, Actb, and Tubb3. If this level of significance is changed to *p *< 0.05, an additional gene associated with axonal regeneration, Stmn2 (SCG-10), achieves significance. These genes are among those up-regulated within PNS neurons during regeneration following a peripheral nerve injury [43], up-regulated within CNS neurons after axotomy close to the cell body [7,8,44], and up-regulated within CNS neurons having axons regenerating into peripheral nerve grafts [7].

However, unlike the PNS where these RAGs remain up-regulated until regenerating axons reach their peripheral target, all of these genes are down-regulated rapidly by 1-week p.i., and approach control levels at longer survival times. This early transient but abortive regenerative response has been reported for particular classes of neurons within the CNS [45-47]. An early regenerative response in corticospinal tract (CST) neurons is seen after axotomy near the cell body, but is not seen after spinal axotomy [8]. An early regenerative response is also seen within rubrospinal tract (RST) neurons after cervical, but not after thoracic axotomy [7]. This difference in the regenerative response is likely the reason RST axons will regenerate within peripheral nerve grafts implanted into the cervical, but not thoracic spinal cord. While there is a large body of supportive evidence that axotomy near the cell body is a key factor in eliciting a regenerative response, there are also exceptions such as Purkinje cells [48]. 2005). The reasons for this difference are unknown except for the possibility that the recurrent collaterals of Purkinje cells are "sustaining", minimizing a cellular reaction [49]. The local axotomy that occurs in TPS neurons after thoracic SCI is a likely reason for the early robust regenerative response found in the present study. This early regenerative response is also likely to be the reason propriospinal axons are better than supraspinal axons in their ability to grow within peripheral nerve or Schwann cell grafts implanted within the spinal cord [9-13]. It is presently unknown if factors in these grafts maintain the cellular response, and/or factors within the CNS environment inhibit it. Schwann cells and vascular macrophages within peripheral nerve grafts secrete a variety of neurotrophic and growth factors as well as extracellular matrix molecules that are both neuroprotective and support continued axonal growth [50]. The factors present in the post-injury spinal cord environment that inhibit axonal regrowth have been extensively reviewed [1,2] and will not be discussed here, but include chondroitin sulfate proteoglycans (CSPGs) and myelin debris, among others.

A recent study conducted by Fenrich and Rose [51] found that, following a focal midline sagittal surgical injury, local propriospinal commissural (PCI) axons regenerate across the lesion site forming specific synaptic connections with contralateral motoneurons. Unlike the present study, PCI neurons did not appear to undergo apoptosis following this injury (see section 3, below). The authors hypothesize that PCI axons are able to respond to guidance molecules in a way that allows them to cross the midline and grow through the CSPG rich lesion site. The findings from the present study, demonstrating a significant down-regulation in the gene for Unc5a, a receptor which mediates the chemorepulsive effect of netrin-1 during development [52], could be related to the success of regrowth, as could the down-regulation of both Lingo1 [27,53] and Ngfrap1[54], receptors that mediate inhibitory cues from the post-injury environment. Previous studies demonstrate that antagonizing Lingo1 or Unc5a, enhances axonal regeneration in the post-lesion environment [27,28,53,55].

Besides the transient up-regulation of RAGs 3-days post-SCI, the largest changes found for most classes of genes occurred during this early period including genes related to the immune and inflammatory response, cell survival, and cell death. Again, these changes returned towards pre-axotomy levels at longer survival times. In the sections below we discuss how (if) the abortive regenerative response of TPS neurons may be related to the timing and transient nature of the immune/inflammatory reaction, or to the apparent cell death of most TPS neurons.

#### Early Inflammatory Response

The present investigation did not examine the genetic response of axotomized TPS neurons during the first 48-hours post p.i. or the early response to pro-inflammatory cytokines which has been described by others following traumatic spinal injury [15]. The genome-wide analysis performed in the present study identified a large number of highly up-regulated genes related to the immune/inflammatory response or cell stress/neuroprotection at the earliest time point, 3-days p.i (Figure. 2, Table 2). The IPA results also showed the three main pathways/networks significantly changed in TPS neurons at this time were related to the inflammatory/immune-response, which was not the case at later periods (Additional File 2; Supplement Table 5). The inflammatory/immune response genes most highly up-regulated at 3-days p.i. returned towards normal by 1-week post-SCI, just as seen for the RAGs.

It is possible that the early robust immune/inflammatory response we observed in our analysis was caused by immune cell contamination acquired during the laser microdissection. Microglial/macrophage contamination cannot be completely excluded since genes expressed by these cells such as Itgam (Integrin alpha M), a.k.a, Cr3 or Cd11b http://www.genecards.org/cgi-bin/carddisp.pl?gene=ITGAM, appeared in our analysis. However, a significant number of contaminating immune cells would have resulted in the up-regulation of several other genes/markers for microglia or macrophages (i.e., Cd163, or Aif1) that was not seen. Furthering our belief that the mRNA collected was from a relatively pure sample of TPS neurons was the lack of expression for Gfap (a marker for astrocytes) and the myelin proteins Mag, Mog, Mbp (markers for both oligodendrocytes and myelin debris). Thus, our data suggests specificity at a cellular rather than a tissue level, indicating mainly intra-neuronal TPS genes have been analyzed.

There is strong evidence that indicates the inflammatory response exacerbates damage at the injury site and contributes to the lack of axonal regeneration, as well as to neuronal apoptosis at the lesion site [15,56]. Therefore, inflammation early post-SCI may be a contributing factor in potential TPS apoptosis (see below). However, other evidence indicates that the inflammatory response and invasion of vascular macrophages [6,38,57-62] may be neuroprotective, as well as stimulating a maximal regenerative response [61,62], and can foster successful regeneration within PNS implants [45-47] or within the CNS, itself [38]. For instance, an inflammatory reaction near their cell bodies increases the expression of RAGs in CST neurons although this response does not contribute to the sprouting or regeneration of CST axons damaged in the spinal cord [38]. In this instance, it would be interesting to see if the damaged CST axons would now be able to regenerate within a peripheral nerve implant. Similarly, retinal ganglion cell axons grow poorly within PNS grafts unless the axons are damaged close to the optic disc, near their cell bodies of origin [63], but regenerate most successfully within peripheral nerve grafts and the optic nerve, itself, with the addition of an inflammatory response resulting from lens injury or other perturbation [58,60]. These studies argue for a component of the inflammatory response playing a major role in the ability of CNS neurons to mount a regenerative response, and may be one reason why injury (axotomy) near the cell body, as in the present case, elicits a regenerative response. Recent evidence suggests that the type of macrophage that may be neuroprotective and supports axonal regeneration is found mainly during the first few days following SCI, and the type of macrophage detrimental to this process is found chronically after SCI [64]. Whether this difference in the inflammatory response is responsible for the time course of the TPS regenerative response remains to be determined.

### 3. Post-axotomy cell death of axotomized TPS neurons?

Preliminary results from our laboratory [17] show a large loss in the number of labeled TPS neurons by 2-weeks p.i. as assessed by first retrogradely pre-labeling these neurons with FG from the lumbosacral enlargement prior to moderate spinal contusion injury. Cell counts 2-weeks p.i. are approximately 70% lower than in uninjured controls. To determine if our retrograde findings were related to cell death, we studied both pro- and anti-apoptotic genes post-SCI in the present analysis. We found a significant up-regulation in gene expression for a number of pro-apoptotic genes including Casp3 (validated with IHC) and no significant change in expression for any anti-apoptotic genes indicating that significant apoptosis is likely to occur post-axotomy. IPA analysis also indicated an early up-regulation of a number of genes involved in apoptosis that returned to control level, or were down-regulated at later time points. The question remains as to whether this change in genetic response of TPS neurons apparent at the end of the first post-operative week resulted from the inability of TPS axons to regenerate in the post-injury spinal environment, or is due to the post-SCI cell death of the majority of axotomized TPS neurons. It may be that the surviving TPS neurons sampled at later survival times are a different population, for instance cells with sustaining collaterals that could limit regeneration and be more resistant to apoptosis.

Again, as with the inflammatory response, the maximal response of RAGs and CsNp genes overlapped with the early up-regulation of pro-apoptotic genes. Others have also reported a parallel increase in a regenerative response post-axotomy, such as an upregulation of Gap43 and an up-regulation of genes involved in apoptosis, usually when axotomy is close to the cell body, and where most axotomized neurons subsequently undergo cell death [65-67]. The post-axotomy apoptosis of neurons in these instances has been prevented or delayed by providing appropriate trophic support [11,12], by placing the damaged axons in an environment that supports/permits axonal regeneration [10,13], or by blocking the apoptotic response [65-67]. Classes of neuron such as the Purkinje cell of the cerebellum normally do not have a regenerative response even with axotomy close to the cell body, and are resistant to post-axotomy cell death [48]. However, in transgenic mice where Gap43 is overexpressed in Purkinje cells, even though axonal sprouting occurs, axotomy leads to cell loss unless these neurons are protected by factors in embryonic grafts placed near the severed axons, as reviewed in [48]. However, in this and other cases [48], even when apoptosis is prevented, significant axonal regeneration does not take place without additional interventions.

### Therapeutic Implications for TPS Neurons

The robust regenerative response of TPS neurons after thoracic SCI is different than that found for most supraspinal neurons (e.g. CST, RST). However, the apparent cell death of the majority of TPS neurons early after thoracic SCI as well as the transient nature of the regenerative response is also different from that found for most supraspinal neurons (CST, RST) where neuronal atrophy results, and cell loss occurs slowly, if at all [68-70]. Previous evidence indicates that propriospinal axons have an advantage over supraspinal axons in their ability to regenerate after SCI [4,11,31,32]. In all of these instances, the affected propriospinal neurons were close to the site of injury, and regenerated into environments conducive to regrowth such as peripheral nerve implants or other type of implant that contained trophic molecules likely to be neuroprotective as well as maintaining the regenerative response. Our findings are most similar to the effects of optic nerve damage near the retina, and the consequent early regenerative and cell death response of the majority of retinal ganglion cells (RGCs). It is possible that treatments enhancing RGC survival and axonal regeneration will also be effective for TPS neurons (potentially including PS neurons locally axotomized by SCI at other spinal levels) including the effects of lens injury that increase RGC survival and axonal regeneration after optic nerve injury [58,60]. Our result indicates that intervention needs to be rapid, within a few days, prior to apoptosis, just as for RGCs. In addition, presenting TPS neurons with specific macrophage related factors may also be beneficial to maintaining axonal regeneration within a CNS environment [64].

To determine the neurotrophic or growth factor(s) that would be most effective in protecting TPS neurons from apoptosis, and could potentially maintain the initial regenerative response, we analyzed surface receptor and growth factor expression profiles (Figure 4B). We found the simultaneous up-regulation of both co-receptor genes for the neurotrophic factor GDNF (Gfra1 and Ret). Iannotti and colleagues [12] have shown that GDNF enhances axonal growth within implants, and, using intrathecal application to the SCI lesion site, is neuroprotective for TPS neurons and several classes of supraspinal neuron post-SCI. In our analysis we also found an upregulation in Lifr, a receptor for LIF and a co-receptor for CNTF. Recently, it was demonstrated that LIF and CNTF are important growth factors caused by lens damage that protect RGCs and stimulate axonal regeneration after optic nerve injury [71]. Other data also suggest that LIF and CNTF might be effective neuroprotective agents for TPS neurons and maintain axonal regeneration after SCI [72-74]. In our analysis we found no significant change in the expression of the Trk receptors for the neurotrophins post-axotomy (NGF, nerve growth factor, Ntrk1 (Trk A); BDNF, brain derived neurotrophic factor, Ntrk2 (Trk B); or NT-3, Ntrk3 (TrkC)). However, the majority of TPS neurons continued to be immunolabeled by appropriate antibodies, suggesting that TPS neurons are likely to remain receptive to these neurotrophins post-SCI. These neurotrophins have been reported to be highly neuroprotective and/or promote axonal sprouting/regeneration in other classes of neuron [4,23,31,32]. Our findings suggest that TPS neurons should be particularly sensitive to GDNF, LIF, and CNTF during the first week p.i., and these are the growth factors most likely to be effective in protecting TPS neurons from apoptosis and maintaining a regenerative response within a CNS environment. There is also evidence these molecules as well as neurotrophins may need to be used in combination acting synergistically on different intracellular targets for a maximal neuroprotective/regenerative response [75].

## Conclusion

Previous studies have demonstrated the ability of PS axons to grow into peripheral nerve implants and neurotrophin enriched bridges, form functional new neuronal bypass circuits around an incomplete lesion, and cross the midline to form new circuits [11,12,47-49]. Our microarray, qRT-PCR, and immunofluoresence data show that TPS neurons respond to axotomy and low thoracic SCI with an early strong inflammatory response and a robust but transient neuroprotective/regenerative response that diminish by the end of the first week p.i., as well as a strong apoptotic response that appears to lead to the loss of the majority of these neurons by 2-weeks p.i.. Early changes in TPS neurons return to near normal levels of expression in 2-week and 4-week samples, suggesting that the surviving TPS neurons sampled at these time points could be a different population, potentially cells with sustaining collaterals that limit the effects of axotomy. Up-regulation of receptors to several growth factors suggest reasons why TPS neurons survive and regenerate within peripheral nerve implants. These specific growth factors could be applied singly or together in future experiments attempting to protect axotomized PS neurons from apoptosis and maintain the regenerative response after spinal cord injury.

## Authors' contributions

DJS and FAM designed the study, JRS performed all microarray and qRT-PCR experiments. JRS, FAM, and DJS all participated in data analysis and interpretation, and JRS wrote the initial manuscript. JRS, FAM, and DJS all reviewed edited and approved the final version of this manuscript.

## Supplementary Material

Additional file 1 **Complete list of genes with the largest significant increases in expression and the largest significant decreases in expression at one or more of the four time-points**. Genes are ranked by their maximal log2 change at one or more of the four time-points. Click here for file

Additional file 2 **The top Bio Functions results were complied from each of the IPA analyses (see Supplemental Data) for all gene networks containing 5 or more genes**. Click here for file

Additional file 3 **Ingenuity Pathway Assist (IPA) analysis of significant genes involved in the inflammatory response**. See text for details. For conventions and symbols, refer to Ingenuity website legend description https://analysis.ingenuity.com/pa/info/help/help.htm#legend.htm. Click here for file

Additional file 4 **Ingenuity Pathway Assist (IPA) analysis of significant genes involved in the cell death response**. See text for details. For conventions and symbols, refer to Ingenuity website legend description https://analysis.ingenuity.com/pa/info/help/help.htm#legend.htm Click here for file

Additional file 5 **Ingenuity Pathway Assist (IPA) analysis of significant genes involved in nervous system development and function**. See text for details. For conventions and symbols, refer to Ingenuity website legend description https://analysis.ingenuity.com/pa/info/help/help.htm#legend.htm. Click here for file

Additional file 6 **Ingenuity Pathway Assist (IPA) analysis of significant genes involved in neurological disease**. See text for details. For conventions and symbols, refer to Ingenuity website legend description https://analysis.ingenuity.com/pa/info/help/help.htm#legend.htm Click here for file

Additional file 7 **Ingenuity Pathway Assist (IPA) analysis of significant genes involved in cell growth and proliferation**. See text for details. For conventions and symbols, refer to Ingenuity website legend description https://analysis.ingenuity.com/pa/info/help/help.htm#legend.htm. Click here for file

Additional file 8 **Complete list of genes compiled for the Regeneration Associated Genes (RAGS) and Cell Survival and Neuroprotection (CsNp) gene programs**. Click here for file

Additional file 9 **Complete list of genes compiled for the Surface Receptor and Growth Factor (GF) gene programs**. Click here for file

Additional file 10 **Complete list of genes compiled for the apoptosis and cell death gene programs**. Click here for file
