# Supplementary material for: Intrinsic response of thoracic propriospinal neurons to axotomy
Source: BMC Neurosci. 2010 Jun 4;11:69. doi: 10.1186/1471-2202-11-69 (PMC2894843; doi:10.1186/1471-2202-11-69)
Supplement: Additional file 1 — Complete list of genes with the largest significant increases in expression and the largest significant decreases in expression at one or more of the four time-points. Genes are ranked by their maximal log2 change at one or more of the four time-points. [file 1471-2202-11-69-S1.PDF]

Additional File 1

Probe ID	Gene Symbol	Gene Title	Max Log2 Δ	Min P-value
		**UPREGULATED FOLLOWING INJURY**		
10775731	Cxcl13	chemokine (C-X-C motif) ligand 13 .	2.88	0.0000176
10728924	Ms4a6b	membrane-spanning 4-domains, subfamily A, member 6B	2.57	0.0015803
10761128	Hspb1	heat shock protein 1	2.50	0.0001084
10821016	Ccnb1	cyclin B1	2.43	0.0020614
10779673	Lgals3	lectin, galactose binding, soluble 3	2.33	0.0073415
10804463	Lox	lysyl oxidase	2.32	0.0013907
10770710	Atf3	activating transcription factor 3	2.26	0.0000860
10720565	Hcst	hematopoietic cell signal transducer	2.22	0.0000348
10931222	Emr1	EGF-like module containing, mucin-like, hormone receptor-like sequence 1	2.06	0.0068668
10710627	Plk1	polo-like kinase 1	2.02	0.0050243
10813392	Fyb	FYN binding protein (FYB-120/130)	2.00	0.0039220
10769657	Nuf2	NUF2, NDC80 kinetochore complex component, homolog	2.00	0.0066665
10785479	Diaph3	similar to Protein diaphanous homolog 3	1.99	0.0031928
10865329	Apobec1	apolipoprotein B mRNA editing enzyme, catalytic polypeptide 1	1.98	0.0011849
10844331	Lcn2	lipocalin 2	1.97	0.0089548
10711268	Itgam	integrin alpha M	1.95	0.0003028
10814430	Cp	ceruloplasmin	1.93	0.0089829
10865336	---	cdna:known chromosome:RGSC3.4:4:159059288:159059386:-1	1.90	0.0040191
10728876	---	cdna:novel chromosome:RGSC3.4:1:213739715:213747087:-1	1.90	0.0056649
10769771	Fcgr2b	Fc fragment of IgG, low affinity IIb, receptor (CD32)	1.89	0.0006027
10936899	Cybb	cytochrome b-245, beta polypeptide	1.85	0.0059066
10838733	Casc5	similar to cancer susceptibility candidate 5 isoform 1	1.83	0.0010235
10858559	Clec4a3	C-type lectin domain family 4, member a3	1.82	0.0013581
10886621	Serpina3n	serine (or cysteine) peptidase inhibitor, clade A, member 3N	1.80	0.0023328
10765850	Spta1	spectrin, alpha, erythrocytic 1	1.77	0.0079428
10933345	Tlr7	toll-like receptor 7	1.75	0.0057452
10819825	---	cdna:novel chromosome:RGSC3.4:2:251238495:251239674:1	1.64	0.0062236
10898192	Tspo	translocator protein	1.64	0.0004559
10880731	C1qc	complement component 1, q subcomponent, C chain	1.63	0.0076456
10751295	Pla1a	phospholipase A1 member A	1.57	0.0010155
10817183	S100a11	S100 calcium binding protein A11 (calizzarin)	1.56	0.0066807
10737663	Gngt2	similar to guanine nucleotide binding protein (G protein), gamma transducing activity polypeptide 2	1.54	0.0046211
10861140	Tfec	transcription factor EC	1.54	0.0076029
10936086	Flna	filamin alpha (actin binding protein 280)	1.53	0.0021075
10933920	RGD1565785	similar to chromosome X open reading frame 21	1.53	0.0045260
10882761	Kpna2	karyopherin (importin) alpha 2	1.52	0.0098349
10860900	Pdk4	pyruvate dehydrogenase kinase, isozyme 4	1.52	0.0033418
10865585	Cd4	Cd4 molecule	1.51	0.0001330
10864918	Ret	ret proto-oncogene	1.50	0.0019093
		**DOWN-REGULATED FOLLOWING INJURY**		
10803692	Nrep	neuronal regeneration related protein	-1.70	0.0003921
10819825	---	cdna:novel chromosome:RGSC3.4:2:251238495:251239674:1	-1.43	0.0062236
10857916	Slc6a11	solute carrier family 6 (neurotransmitter transporter, GABA), member 11	-1.40	0.0003994
10795921	---	cdna:pseudogene chromosome:RGSC3.4:17:71717990:71718317:1	-1.37	0.0084528
10888931	Abhd1	abhydrolase domain containing 1	-1.19	0.0058983
10862194	LOC679835	similar to Anionic trypsin II precursor	-1.18	0.0012800
10722918	Rlbp1	retinaldehyde binding protein 1	-1.14	0.0070503
10825100	Car14	carbonic anhydrase 14 (Car14)	-1.12	0.0094842
10758031	---	ncrna:snoRNA chromosome:RGSC3.4:12:27994393:27994527:1	-1.02	0.0013763
10923177	---	cdna:novel chromosome:RGSC3.4:9:45558789:45559112:1	-1.02	0.0023602
10905284	Tst	thiosulfate sulfurtransferase	-1.00	0.0006739
10777123	---	cdna:known chromosome:RGSC3.4:14:66133358:66133777:-1	-1.00	0.0031577
10815074	---	ncrna:snRNA chromosome:RGSC3.4:2:126623221:126623376:1	-0.92	0.0056085
10872473	Fabp3	fatty acid binding protein 3, muscle and heart	-0.89	0.0024797
10765740	Kcnj10	potassium inwardly-rectifying channel, subfamily J, member 10	-0.86	0.0028366
10722513	---	ncrna:miRNA chromosome:RGSC3.4:1:115856385:115856462:-1	-0.85	0.0052890
10940654	---	secreted phosphoprotein 1	-0.84	0.0019474
10749975	---	MI0000883 miR-99a stem-loop	-0.84	0.0059620
10844268	---	ncrna:snRNA chromosome:RGSC3.4:3:10121982:10122132:-1	-0.83	0.0077416
10742645	Slc22a5	solute carrier family 22 (organic cation/carnitine transporter), member 5	-0.83	0.0004709
10893412	Olr1063	olfactory receptor 1063	-0.83	0.0016334
10782144	Clybl	citrate lyase beta like	-0.82	0.0090062
10906608	Slc38a2	solute carrier family 38, member 2	-0.81	0.0041913
10706134	Slc7a10	solute carrier family 7, (neutral amino acid transporter, y+ system) member 10	-0.80	0.0031830
10805605	Dok6	similar to docking protein 5-like	-0.80	0.0046691
10896666	RGD1311362	similar to hypothetical protein FLJ10204	-0.79	0.0076024
10822735	Pex5l	peroxisomal biogenesis factor 5-like	-0.78	0.0032250
10785773	Spry2	sprouty homolog 2	-0.77	0.0041099
10769215	---	cdna:novel chromosome:RGSC3.4:13:76730411:76732774:-1	-0.73	0.0041856
10721796	Lin7b	lin-7 homolog b	-0.72	0.0044215
10940566	---	common salivary protein 1	-0.72	0.0055240
10878210	Kank4	KN motif and ankyrin repeat domains 4	-0.72	0.0036238
10854847	---	cdna:novel chromosome:RGSC3.4:4:68287242:68296614:1	-0.71	0.0073352
10862798	Fam13a1	similar to family with sequence similarity 13, member A1	-0.71	0.0032615
10861358	Gpr37	G protein-coupled receptor 37	-0.71	0.0033955
10869339	LOC500475	similar to hypothetical protein 4933430I17	-0.71	0.0003324
10896836	Efr3a	EFR3 homolog A	-0.71	0.0021565
10768726	Npl	N-acetylneuraminate pyruvate lyase	-0.70	0.0076349
