# Supplementary material for: Intrinsic response of thoracic propriospinal neurons to axotomy
Source: BMC Neurosci. 2010 Jun 4;11:69. doi: 10.1186/1471-2202-11-69 (PMC2894843; doi:10.1186/1471-2202-11-69)
Supplement: Additional file 2 — The top Bio Functions results were complied from each of the IPA analyses (see Supplemental Data) for all gene networks containing 5 or more genes. [file 1471-2202-11-69-S2.PDF]

Additional File 2

Top Functions Mapped in Significantly Changes Genes (IPA Analysis)

3day post

Category	B-H Min P	B-H Max P	N	Molecules
Antigen Presentation	0.000000	0.095000	52	RAC2,LGALS3,RARRES2,MMP3,CD4,SERPINA3,HEBP1,CD163,FCGR1A,PTPRC,VEGFA,CTSL2,CXCL13,CXCL14,S100A10,PTPN6,C3,DCN,LCN2,ANXA2,IFNGR1,GSN,TLR2,ITGAM,THBS2,CD14,VAV1,IRF8,C3AR1,CD151,FN1,MSR1,FGF2,FCGR2B,CCL13,IGF1,ANXA1,PROS1,TLR7,LBP,AGT,CXCL11,ITGB1,IL21,UCP2,MYD88,TYROBP,ITGAL,INPP5D,GRN,CD44,IL1B
Inflammatory Response	0.000000	0.079400	52	RAC2,RARRES2,LGALS3,MMP3,CD4,SERPINA3,C1QA,HEBP1,CD163,FCGR1A,CTSL2,VEGFA,PTPRC,CXCL13,CXCL14,S100A10,PTPN6,LCP1,C3,DCN,LCN2,IFNGR1,ANXA2,STAT3,GSN,TLR2,CSF2RB,ITGAM,THBS2,CTSB,CD14,VAV1,IRF8,C3AR1,CD151,FN1,MSR1,FGF2,FCGR2B,CCL13,IGF1,PROS1,ANXA1,TLR7,LBP,GJB2,AGT,ITGB1,IL21,UCP2,MYD88,TYROBP,NQO1,ALOX5AP,ITGAL,INPP5D,GRN,LYN,CD44,IL1B
Cell-mediated Immune Response	0.000000	0.103000	44	RAC2,FN1,RARRES2,BDNF,CD4,CD83,SERPINA3,HEBP1,CD163,VEGFA,CTSL2,CCL13,IGF1,CXCL13,PROS1,ANXA1,GFRA1,CXCL14,TLR7,LBP,GJB2,S100A10,AGT,IL21,ITGB1,PTPN6,C3,MYD88,LCN2,ALOX5AP,ANXA2,STAT3,GSN,ITGAL,TLR2,ITGAM,LYN,CD44,CTSB,CD14,IL1B,VAV1,LCP2,CD151
Humoral Immune Response	0.000000	0.000006	31	TLR7,LBP,S100A10,ITGB1,IL21,PTPN6,C3,MYD88,LCN2,ANXA2,ITGAL,TLR2,ITGAM,CD14,IL1B,VAV1,CD151
Cell-To-Cell Signaling and Interaction	0.000002	0.084500	52	RARRES2,CD4,CD163,FCGR1A,PTPRC,VEGFA,CXCL14,S100A10,ATM,PTPN6,LCP1,C3,DCN,LCN2,ANXA2,STAT3,TLR2,CSF2RB,ITGAM,KCNJ10,THBS2,CD14,VAV1,C3AR1,FN1,MSR1,BDNF,FGF2,CD83,FCGR2B,HMOX1,CCL13,IGF1,GFRA1,ANXA1,PROS1,TLR7,LBP,CXCL11,AGT,ITGB1,IL21,UCP2,MYD88,ITGAL,INPP5D,CLEC7A,CDKN1A,CD44,LYN,IL1B,HTR1A
Hematological System Development and Function	0.000002	0.095000	52	L13,CXCL14,S100A10,LCP1,PTPN6,C3,DCN,LCN2,IFNGR1,ANXA2,STAT3,GSN,TLR2,CSF2RB,ITGAM,THBS2,CTSB,CD14,VAV1,IRF8,C3AR1,LCP2,CD151,FN1,MSR1,FGF2,CD83,FCGR2B,CCL13,IGF1,PROS1,ANXA1,TLR7,LBP,GJB2,AGT,IL21,ITGB1,UCP2,TYROBP,MYD88,NQO1,ALOX5AP,ITGAL,INPP5D,GRN,LYN,CD44,IL1B
Immune Cell Trafficking	0.000006	0.095000	52	RAC2,RARRES2,LGALS3,MMP3,CD4,SERPINA3,HEBP1,CD163,CTSL2,VEGFA,PTPRC,CXCL13,CXCL14,S100A10,PTPN6,LCP1,C3,LCN2,ANXA2,STAT3,GSN,TLR2,ITGAM,THBS2,CTSB,CD14,VAV1,C3AR1,CD151,FN1,MSR1,FGF2,FCGR2B,CCL13,IGF1,ANXA1,PROS1,TLR7,LBP,GJB2,CXCL11,AGT,ITGB1,IL21,UCP2,MYD88,ALOX5AP,ITGAL,INPP5D,GRN,CD44,LYN,IL1B
Cell Death	0.000009	0.095000	31	LGALS3,MSR1,BDNF,FGF2,PTPRC,VEGFA,CTSL2,CCL13,IGF1,ANXA1,CYBB (includes EG:1536),NLRC4,ATM,UCP2,CASP3,MYD88,DCN,STAT3,GSN,CDC2,INPP5D,TLR2,CSF2RB,PRKCD,ATP1A2,CDKN1A,LYN,CTSB,CD14,IL1B,HSPB1
Cellular Movement	0.000010	0.095000	34	RAC2,RARRES2,LGALS3,FN1,MSR1,BDNF,SERPINA3,HEBP1,CTSL2,VEGFA,DAO,CCL13,CXCL13,ANXA1,GFRA1,CXCL14,GJB2,CXCL11,AGT,ITGB1,PTPN6,C3,MYD88,ALOX5AP,STAT3,GSN,ITGAL,TLR2,ITGAM,NDE1,CTSB,CD44,IL1B,CD151
Tissue Morphology	0.000016	0.077000	28	RAC2,LGALS3,MMP3,FGF2,BDNF,CD4,C1QA,FCGR1A,PGR,CTSL2,VEGFA,CCL13,IGF1,GFRA1,ANXA1,CYBB (includes EG:1536),ATM,AGT,CASP3,MYD88,TYROBP,NQO1,IFNGR1,INPP5D,KCNJ10,NDE1,IL1B,IRF8
Immunological Disease	0.000024	0.077000	24	MME,LGALS3,MSR1,CASP3,MYD88,DCN,HSPB8,CD83,ANXA2,STAT3,IL17RA,GSN,INPP5D,PTPRC,TLR2,NR4A2,ITGAM,ANXA1,PRKCD,CDKN1A,CD14,IL1B,NLRC4,S100A10
Hematological Disease	0.000024	0.077000	18	LGALS3,MSR1,CASP3,MYD88,DCN,ANXA2,STAT3,GSN,INPP5D,PTPRC,TLR2,ITGAM,ANXA1,PRKCD,CDKN1A,CD14,IL1B,NLRC4
Tissue Development	0.000114	0.077000	22	ITGB1,LCP1,ATF3,FN1,MMP3,MYD88,BDNF,FGF2,CD4,STAT3,ITGAL,INPP5D,GRN,VEGFA,CCL13,ITGAM,ANXA1,CD44,LYN,IL1B,VAV1,AGT
Skeletal and Muscular Disorders	0.000187	0.077000	18	MME,BDNF,HSPB8,VIM,SERPINA3,CD83,ANXA2,STAT3,IL17RA,ARHGDIB,TUBB2B,TLR2,PTPRC,NCF1,NR4A2,IL1B,LDHA,S100A10
Cellular Function and Maintenance	0.000281	0.077000	18	ITGB1,FN1,C3,TYROBP,FGF2,BDNF,STAT3,FCGR2B,ITGAL,INPP5D,VEGFA,CSF2RB,ITGAM,ANXA1,CD44,LYN,IRF8,ATM
Inflammatory Disease	0.001050	0.077000	12	MME,PTPRC,TLR2,NCF1,NR4A2,HSPB8,IL1B,CD83,EMR1,STAT3,IL17RA,S100A10
Genetic Disorder	0.001480	0.066700	28	MME,RIT2,BDNF,HSPB8,CD83,SERPINA3,IL17RA,TUBB2B,PTPRC,GPR37,S100A10,CALY,CASP3,TXNIP,TYROBP,NQO1,VIM,ANXA2,GSN,ARHGDIB,TLR2,NR4A2,PRKAR2B,IL1B,EMR1,MMAA,C5ORF13,LDHA
Connective Tissue Disorders	0.001620	0.077000	11	MME,PTPRC,TLR2,NCF1,NR4A2,HSPB8,IL1B,CD83,STAT3,IL17RA,S100A10
Neurological Disease	0.006570	0.103000	30	MME,BDNF,RIT2,SERPINA3,TUBB2B,VEGFA,CTSL2,IGF1,GPR37,CYBB (includes EG:1536),PGDS,CALY,ATM,UCP2,CASP3,TXNIP,TYROBP,NQO1,VIM,ANXA2,GSN,CDC2,ARHGDIB,PRKAR2B,ATP1A2,CTSB,IL1B,MMAA,C5ORF13,LDHA
Cellular Development	0.006600	0.079400	23	IL21,FN1,BDNF,MYD88,FGF2,CD4,C1QC,STAT3,INPP5D,VEGFA,CSF2RB,CCL13,RUNX1T1,ITGAM,IGF1,TIMP1,GFRA1,TLR7,LYN,IL1B,IRF8,LCP2,CXCL11
Nervous System Development and Function	0.008170	0.103000	27	FN1,FGF2,BDNF,SGK1,VEGFA,DAO,HTR1B,IGF1,BCAN,GFRA1,CYBB (includes EG:1536),AGT,ATM,ITGB1,ATF3,NCAN,STAT3,NCF1,NDE1,KCNJ10,CDKN1A,IL1B,HTR1A,SFRP1,A2M,HSPB1,NRP1
Cell Morphology	0.008170	0.103000	21	ITGB1,FN1,BDNF,FGF2,STAT3,VEGFA,CCL13,HTR1B,ITGAM,IGF1,KCNJ10,FLNA,BCAN,ANXA1,CDKN1A,CD44,LYN,SFRP1,A2M,NRP1,ATM
Cellular Assembly and Organization	0.008170	0.077000	19	ITGB1,UCP2,FN1,BDNF,FGF2,SERPINA3,STAT3,VEGFA,ITGAM,HTR1B,IGF1,NDE1,BCAN,GFRA1,CDKN1A,IL1B,HTR1A,A2M,NRP1
Cellular Growth and Proliferation	0.008980	0.079400	16	IL21,CASP3,BDNF,FGF2,CD4,DCN,CDKN2C,CD83,VEGFA,IGF1,CDKN1A,IL1B,HTR1A,IRF8,LCP2,ATM
Free Radical Scavenging	0.008980	0.008980	5	RAC2,NCF1,ITGAM,CYBB (includes EG:1536),AGT
Cell Signaling	0.016800	0.079400	22	UCP2,RARRES2,MYD88,BDNF,FGF2,S100A4,SERPINA3,HEBP1,TLR2,PTPRC,ITGAM,CCL13,CXCL13,PRKCD,ANXA1,IL1B,ST14,PTAFR,A2M,LCP2,AGT,CXCL11
Molecular Transport	0.016800	0.095000	22	RARRES2,MSR1,BDNF,FCRL2,FGF2,NPPC,S100A4,HEBP1,PTPRC,TLR2,HTR1B,CCL13,CXCL13,KCNJ10,ANXA1,IL1B,PTAFR,A2M,LCP2,AGT,ATM,CXCL11
Vitamin and Mineral Metabolism	0.016800	0.079400	17	RARRES2,BDNF,FGF2,S100A4,HEBP1,TLR2,PTPRC,ITGAM,CCL13,CXCL13,ANXA1,IL1B,PTAFR,A2M,LCP2,AGT,CXCL11
Small Molecule Biochemistry	0.022200	0.095000	15	UCP2,MSR1,FGF2,BDNF,FCRL2,MYD88,NPPC,HTR1B,KCNJ10,PRKCD,IL1B,CD14,PTAFR,ST14,ATM
Lipid Metabolism	0.022200	0.077000	6	UCP2,MSR1,FCRL2,BDNF,NPPC,PTAFR
Gene Expression	0.035600	0.077000	7	ITGAM,FN1,BDNF,FGF2,CD4,IL1B,IRF8
Amino Acid Metabolism	0.035600	0.077000	6	FGF2,BDNF,KCNJ10,IL1B,THRB (includes EG:7068),ATM
Cell Cycle	0.035600	0.079400	3	NDE1,DCN,A2M
Gastrointestinal Disease	0.063300	0.077000	3	CDKN1A,EMR1,STAT3
Cellular Compromise	0.077000	0.077000	4	IGF1,MSR1,CD44,ATM
DNA Replication, Recombination, and Repair	0.077000	0.077000	4	NDE1,ATM
Hematopoiesis	0.077000	0.077000	3	IL21,IRF8,STAT3
Behavior	0.077000	0.077000	3	SGK1,BDNF,PGR
Carbohydrate Metabolism	0.077000	0.077000	3	BDNF,CD14,PTAFR

1 wk post

Category	B-H Min P	B-H Max P	N	Molecules
Nervous System Development and Function	0.027200	0.130000	18	IFT88, ATF3, CASP3, EMP3, EGR1, ZIC1, STX6, C19ORF20, NPAS3, INHBA, METRN, DIAPH1, KCNMA1, KCNJ10, SFRP1, ADCYAP1, NRP1, HMGB2
Cellular Growth and Proliferation	0.027200	0.141000	9	IFT88, CASP3, EGR1, CD4, ZIC1, NPAS3, GATA3, ADCYAP1, INHBA
Neurological Disease	0.032200	0.130000	5	IFT88, CASP3, ATXN1, ZIC1, CAST

2 wk post

Category	B-H Min P	B-H Max P	N	Molecules
Hematological System Development and Function	0.120000	0.151000	14	RAC2,PLCB2,LGALS3,MMP3,CD83,CTLA4,CXCL10,HMOX1,B4GALT1,CDKN1A,LSP1,IRF8,NMU,CXCL11
Cellular Movement	0.120000	0.120000	7	CXCL10,RAC2,PLCB2,LGALS3,B4GALT1,NMU,CXCL11
Inflammatory Response	0.120000	0.151000	7	CXCL10,RAC2,PLCB2,B4GALT1,CDKN1A,NMU,CXCL11
Cell-To-Cell Signaling and Interaction	0.120000	0.151000	6	CXCL10,HMOX1,CDKN1A,CD83,CTLA4,CXCL11
Immune Cell Trafficking	0.120000	0.120000	6	CXCL10,RAC2,PLCB2,B4GALT1,NMU,CXCL11
Nervous System Development and Function	0.120000	0.151000	6	GAL,SGK1,CDKN1A,PAX6,A2M,CBR1
Tissue Morphology	0.120000	0.151000	6	CXCL10,LGALS3,MMP3,LSP1,PAX6,IRF8
Cellular Growth and Proliferation	0.120000	0.151000	5	CDKN1A,PAX6,CD83,CTLA4,CBR1
Endocrine System Development and Function	0.120000	0.151000	4	ABCB1,PAX6,HES1,NMU
Behavior	0.120000	0.151000	3	GAL,SGK1,NMU
Cell-mediated Immune Response	0.120000	0.151000	3	HMOX1,CD83,CTLA4
Antigen Presentation	0.120000	0.151000	3	CXCL10,A2M,CXCL11

1 mo post

Category	B-H Min P	B-H Max P	N	Molecules
Nervous System Development and Function	0.046700	0.118000	14	NPY, ATF3, BDNF, EGR1, CRH, BAX, BCL2, PICK1, JUN, GAL, IGF1, NR3C2, ADCYAP1, HSPB1
Neurological Disease	0.046700	0.086200	10	ABCD2, IL18, JUN, IGF1, GAL, BDNF, ITGB8, BAX, ADCYAP1, BCL2
Cell Death	0.046700	0.086200	9	ABCD2, JUN, IGF1, GAL, BDNF, BAX, ADCYAP1, HSPB1, BCL2
Behavior	0.046700	0.102000	5	NPY, JUN, GAL, BDNF, CRH
Digestive System Development and Function	0.046700	0.046700	3	NPY, GAL, CRH
Cellular Development	0.051200	0.118000	17	STAT6, THPO, BDNF, EGR1, XBP1, C1QC, BAX, BCL2, TRAF6, IL18, JUN, IGF1, TIMP1, PLCG2, CORO1A, CEBPA, ADCYAP1
Hematological System Development and Function	0.051200	0.118000	17	STAT6, THPO, TYROBP, EGR1, XBP1, C1QC, P2RX7, CD74, BCL2, TRAF6, IL18, IGF1, TIMP1, PLCG2, CEBPA, ADCYAP1, HLA-C
Hematopoiesis	0.051200	0.104000	11	TRAF6, STAT6, THPO, IL18, TIMP1, EGR1, PLCG2, CEBPA, XBP1, C1QC, BCL2
Cell-To-Cell Signaling and Interaction	0.067600	0.118000	7	IL18, PICK1, GAL, IGF1, BDNF, P2RX7, HLA-C
Tissue Development	0.067600	0.086200	3	ATF3, BDNF, BAX
Inflammatory Response	0.084800	0.086200	8	IL18, C3, IGF1, TYROBP, XBP1, P2RX7, MAPKAPK2, ADCYAP1
Cellular Function and Maintenance	0.084800	0.117000	7	STAT6, IL18, C3, BDNF, TYROBP, CRH, ADCYAP1
Immunological Disease	0.084800	0.118000	7	STAT6, P2RY13, BTC, CEBPA, CD74, S100A10, BCL2
Cancer	0.084800	0.086200	3	TIMP1, CEBPA, BCL2
Cell Morphology	0.086200	0.118000	11	STAT6, IL18, JUN, GAL, BDNF, EGR1, CORO1A, CRH, NR3C2, CD74, ADCYAP1
Cellular Growth and Proliferation	0.086200	0.118000	10	STAT6, IL18, THPO, IGF1, BDNF, EGR1, BAX, MAPKAPK2, ADCYAP1, HLA-C
Tissue Morphology	0.086200	0.118000	10	STAT6, IGF1, GAL, BDNF, TYROBP, CRH, XBP1, BAX, ADCYAP1, BCL2
Cell-mediated Immune Response	0.086200	0.118000	9	STAT6, IL18, JUN, BDNF, CORO1A, NR3C2, CD74, ADCYAP1, HLA-C
Molecular Transport	0.086200	0.118000	9	ABCD2, NPY, IGF1, GAL, BDNF, CRH, SLC11A2, PTAFR, ADCYAP1
Small Molecule Biochemistry	0.086200	0.118000	8	ABCD2, NPY, IGF1, GAL, BDNF, CRH, ADCYAP1, PTAFR
Genetic Disorder	0.086200	0.118000	6	NPY, STAT6, P2RY13, BTC, CD74, S100A10
Cell Signaling	0.086200	0.118000	5	IGF1, BDNF, CRH, PTAFR, ADCYAP1
Antigen Presentation	0.086200	0.086200	4	IGF1, TYROBP, XBP1, P2RX7
Vitamin and Mineral Metabolism	0.086200	0.086200	4	BDNF, CRH, PTAFR, ADCYAP1
Lipid Metabolism	0.086200	0.086200	4	ABCD2, NPY, BDNF, PTAFR
Nucleic Acid Metabolism	0.086200	0.118000	3	IGF1, CRH, ADCYAP1
Amino Acid Metabolism	0.086200	0.086200	3	NPY, GAL, BDNF
Cardiovascular Disease	0.086200	0.086200	3	JUN, IGF1, EGR1
Cellular Assembly and Organization	0.086200	0.086200	3	IGF1, CRH, ADCYAP1
Gene Expression	0.086200	0.088600	3	JUN, BDNF, EGR1
Lymphoid Tissue Structure and Development	0.104000	0.104000	3	EGR1, PLCG2, XBP1
Connective Tissue Disorders	0.118000	0.118000	5	STAT6, P2RY13, BTC, CD74, S100A10
Inflammatory Disease	0.118000	0.118000	5	STAT6, P2RY13, BTC, CD74, S100A10
Skeletal and Muscular Disorders	0.118000	0.118000	5	STAT6, P2RY13, BTC, CD74, S100A10
