# Supplementary material for: Intrinsic response of thoracic propriospinal neurons to axotomy
Source: BMC Neurosci. 2010 Jun 4;11:69. doi: 10.1186/1471-2202-11-69 (PMC2894843; doi:10.1186/1471-2202-11-69)

Additional File 5

Nervous System Development and Function

3 Days Post Injury

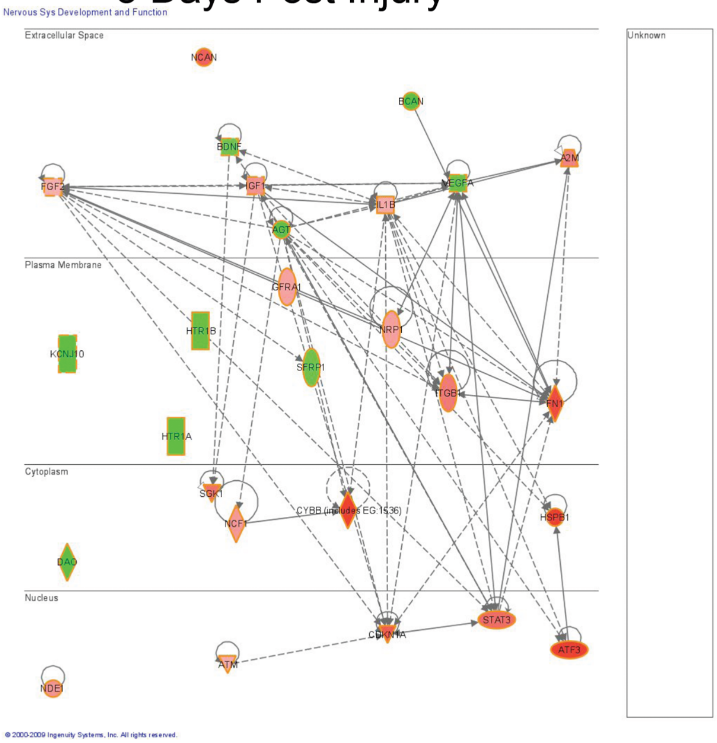

1 Week Post Injury

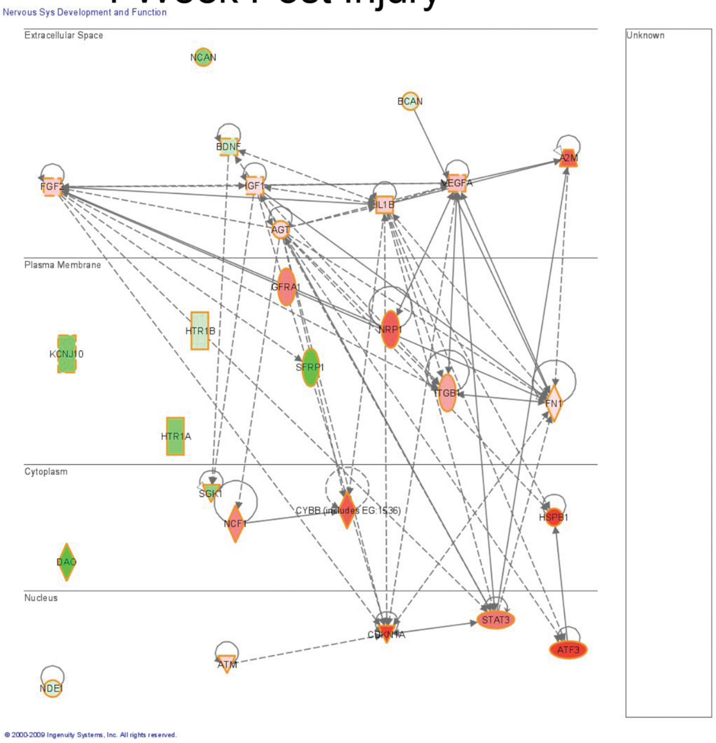

2 Week Post Injury

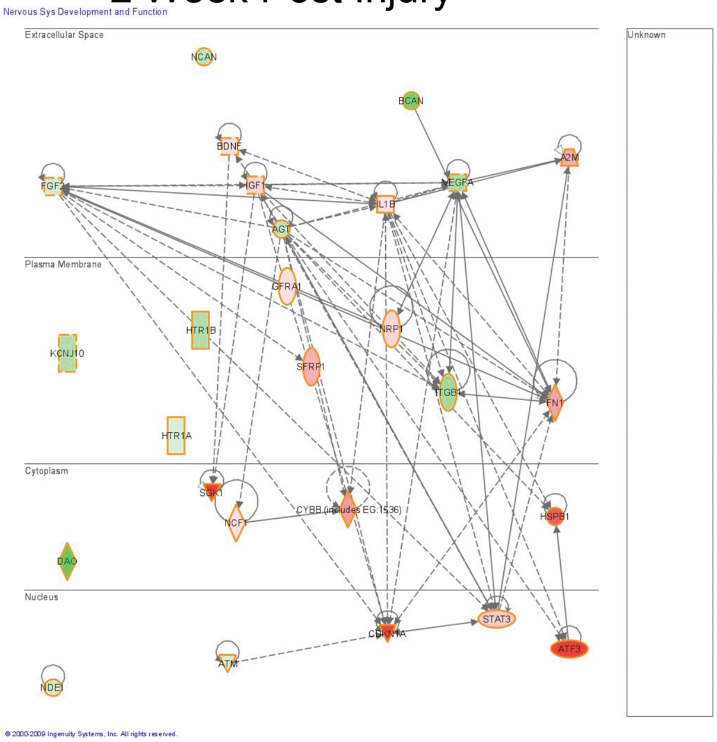

1 Month Post Injury

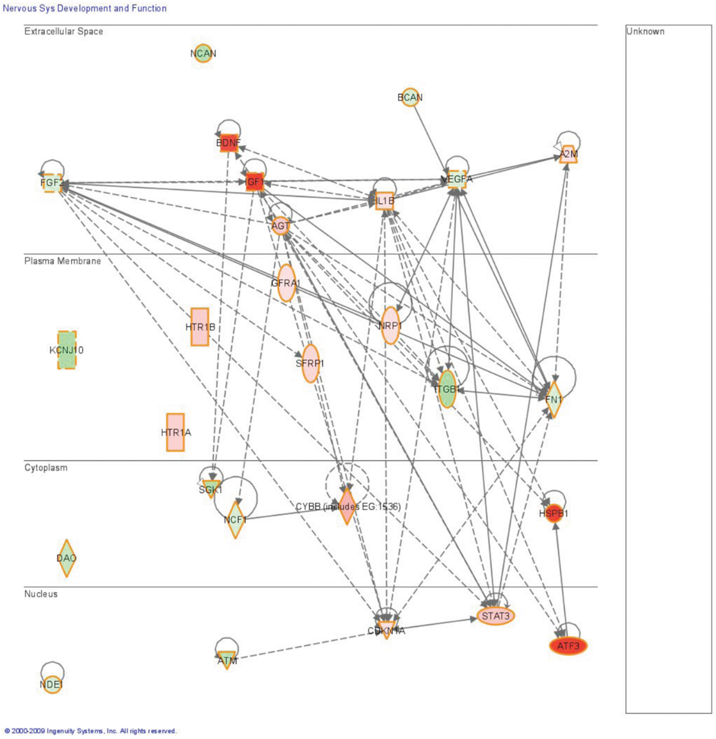

Supplement: Additional file 5 — Ingenuity Pathway Assist (IPA) analysis of significant genes involved in nervous system development and function. See text for details. For conventions and symbols, refer to Ingenuity website legend description https://analysis.ingenuity.com/pa/info/help/help.htm#legend.htm. [file 1471-2202-11-69-S5.PDF]
