# Supplementary material for: Intrinsic response of thoracic propriospinal neurons to axotomy
Source: BMC Neurosci. 2010 Jun 4;11:69. doi: 10.1186/1471-2202-11-69 (PMC2894843; doi:10.1186/1471-2202-11-69)
Supplement: Additional file 8 — Complete list of genes compiled for the Regeneration Associated Genes (RAGS) and Cell Survival and Neuroprotection (CsNp) gene programs. [file 1471-2202-11-69-S8.PDF]

Additional File 8

Regeneration Associated Genes (RAGS) and Cell Survival and Neuroprotective Genes (CsNp)

Probe ID	Gene Symbol	Gene Title
10756799	Actb	actin, beta
10726550	Adam8	a disintegrin and metallopeptidase domain 8
10930539	Adcyap1	adenylate cyclase activating polypeptide 1
10775866	Areg	amphiregulin
10702214	Arg1	arginase 1, liver
10846233	Atf2	activating transcription factor 2
10770710	Atf3	activating transcription factor 3
10839974	Atrn	attractin
10822075	Basp1	brain abundant, membrane attached signal protein 1
10785063	Bmp1	bone morphogenetic protein 1
10857064	Bmp10	bone morphogenetic protein 10
10840138	Bmp2	bone morphogenetic protein 2
10775573	Bmp3	bone morphogenetic protein 3
10782891	Bmp4	bone morphogenetic protein 4
10911711	Bmp5	bone morphogenetic protein 5
10797949	Bmp6	bone morphogenetic protein 6
10852106	Bmp7	bone morphogenetic protein 7
10879547	Bmp8a	bone morphogenetic protein 8a
10734761	Ccdc42	coiled-coil domain containing 42
10847761	Cd44	CD44 antigen
10842469	Cebpb	CCAAT/enhancer binding protein (C/EBP), beta
10712829	Coro1b	coronin, actin-binding protein, 1B
10923929	Creb1	cAMP responsive element binding protein 1
10795616	Crem	cAMP responsive element modulator
10780990	Ctsb	cathepsin B
10800919	Egr1	early growth response 1
10878712	Elavl4	ELAV (embryonic lethal, abnormal vision, Drosophila)-like 4 (Hu antigen D)
10813172	Fgf10	fibroblast growth factor 10
10862867	Gadd45a	growth arrest and DNA-damage-inducible 45 alpha
10727321	Gal	galanin
10739788	Galr2	galanin receptor 2
10751218	Gap43	growth associated protein 43
10781410	Gfra2	glial cell line derived neurotrophic factor family receptor alpha 2
10835775	Gsn	gelsolin
10748962	Hn1	hematological and neurological expressed sequence 1
10761128	Hspb1	heat shock protein 1
10928563	Idh1	isocitrate dehydrogenase 1 (NADP+), soluble
10702996	Igf2r	insulin-like growth factor 2 receptor
10778390	Igfbp3	insulin-like growth factor binding protein 3
10899465	Igfbp6	insulin-like growth factor binding protein 6
10859799	Il6	interleukin 6
10709629	Ilk	integrin linked kinase
10767095	Inhbb	inhibin beta-B
10836849	Itga6	integrin, alpha 6
10893267	Itga7	integrin alpha 7
10812021	Itgb1	integrin beta 1 (fibronectin receptor beta)
10878112	Jun	Jun oncogene (Jun), mRNA.
10940195	L1cam	L1 cell adhesion molecule
10749495	Lgals3bp	lectin, galactoside-binding, soluble, 3 binding protein
10843656	Lhx3	LIM homeobox protein 3
10706320	Lim2	lens intrinsic membrane protein 2
10760646	Mafk	v-maf musculoaponeurotic fibrosarcoma oncogene family, protein K (avian)
10820835	Map1b	microtubule-associated protein 1b
10872336	Marcksl1	MARCKS-like 1
10829649	Mif	macrophage migration inhibitory factor
10839135	Mtap1a	microtubule-associated protein 1 A
10855506	Npy	neuropeptide Y
10883785	Odc1	ornithine decarboxylase 1
10937362	Pak3	p21 (CDKN1A)-activated kinase 3
10908347	Pde4a	phosphodiesterase 4A, cAMP specific
10790921	Pde4c	phosphodiesterase 4C, cAMP specific
10814726	Pik3ca|LOC685590	phosphatidylinositol 3-kinase, catalytic, alpha polypeptide | similar to Phosphatidylinositol-4,5-bisphosphate 3-kinase catalytic subunit alpha isoform (PI3-kinase p110 subunit alpha) (PtdIns-3-kinase p110) (PI3K)
10798096	Psmg4|Pde4b|LOC682709	proteasome (prosome, macropain) assembly chaperone 4 | phosphodiesterase 4B, cAMP specific | hypothetical protein LOC682709
10861835	Ptn	pleiotrophin
10816941	Rab13	RAB13, member RAS oncogene family
10729336	RGD1563350	similar to Fatty acid-binding protein, epidermal (E-FABP)
10882752	Rhoq	ras homolog gene family, member Q
10889415	Sox11	SRY-box containing gene 11
10824732	Sprr1a	small proline-rich protein 1A
10874019	Srm	spermidine synthase
10747506	Stat3	signal transducer and activator of transcription 3
10852531	Stmn3	stathmin-like 3
10781197	Stmn4	stathmin-like 4
10705213	Tgfb1	transforming growth factor, beta 1
10770577	Tgfb2	transforming growth factor, beta 2
10891303	Tgfb3	transforming growth factor, beta 3
10863282	Tmsb10	thymosin, beta 10
10819874	Tnni3k	TNNI3 interacting kinase
10898192	Tspo	translocator protein (Tspo), mRNA.
10907086	Tuba1a	tubulin, alpha 1A
10808702	Tubb3	tubulin, beta 3
10772705	Ugdh	UDP-glucose dehydrogenase
10757599	Ywhag	tyrosine 3-monooxygenase/tryptophan 5-monooxygenase activation protein, gamma polypeptide
10777829	Ywhah	tyrosine 3-monooxygenase/tryptophan 5-monooxygenase activation protein, eta polypeptide
10889360	Ywhaq	tyrosine 3-monooxygenase/tryptophan 5-monooxygenase activation protein, theta polypeptide
