# Supplementary material for: Intrinsic response of thoracic propriospinal neurons to axotomy
Source: BMC Neurosci. 2010 Jun 4;11:69. doi: 10.1186/1471-2202-11-69 (PMC2894843; doi:10.1186/1471-2202-11-69)
Supplement: Additional file 9 — Complete list of genes compiled for the Surface Receptor and Growth Factor (GF) gene programs. [file 1471-2202-11-69-S9.PDF]

Additional File 9

Surface Receptor and Growth Factor (GF) Genes

Probe ID	Gene Symbol	Gene Title
10889513	Acp1	acid phosphatase 1, soluble (Acp1), mRNA.
10890338	Acp1	acid phosphatase 1, soluble (Acp1), mRNA.
10932236	Acp1	acid phosphatase 1, soluble (Acp1), mRNA.
10837832	Acp2	acid phosphatase 2, lysosomal (Acp2), mRNA.
10845429	Acvr1	activin A receptor, type 1 (Acvr1), mRNA.
10899367	Acvr1b	activin A receptor, type 1B (Acvr1b), mRNA.
10845416	Acvr1c	activin A receptor, type IC (Acvr1c), mRNA.
10836072	Acvr2a	activin receptor IIA (Acvr2a), mRNA.
10914230	Acvr2b	activin receptor IIB (Acvr2b), mRNA.
10899354	Acvrl1	activin A receptor type II-like 1 (Acvrl1), mRNA.
10705394	Akt2	thymoma viral proto-oncogene 2 (Akt2), mRNA.
10899554	Amhr2	anti-Mullerian hormone type 2 receptor (Amhr2), mRNA.
10903529	Angpt1	angiopoietin 1 (Angpt1), mRNA.
10789291	Angpt2	cDNA clone MGC:187710 IMAGE:9021835, complete cds.
10755448	Ap2m1	adaptor-related protein complex 2, mu 1 subunit (Ap2m1), mRNA.
10911589	Arpp19	cAMP-regulated phosphoprotein 19 (Arpp19), mRNA.
10920541	Arpp19	cAMP-regulated phosphoprotein 19 (Arpp19), mRNA.
10879084	Artn	artemin (Artn), mRNA.
10899248	Atf1	similar to Cyclic AMP-dependent transcription factor ATF-1 gene:ENSRNOG00000032162
10713222	Atg2a|Ehd1|LOC68485	ATG2 autophagy related 2 homolog A (S. cerevisiae) (Atg2a), mRNA.
10719900	Axl	Axl receptor tyrosine kinase (Axl), transcript variant 1, mRNA.
10811185	Bcar1	breast cancer anti-estrogen resistance 1 (Bcar1), mRNA.
10914799	Birc3	baculoviral IAP repeat-containing 3 (Birc3), mRNA.
10790481	Bmpr1a	bone morphogenetic protein receptor, type 1A (Bmpr1a), mRNA.
10827068	Bmpr1b	bone morphogenetic protein receptor, type 1B (mapped) (Bmpr1b), mRNA.
10923687	Bmpr2	similar to Bone morphogenetic protein receptor type-2 precursor gene:ENSRNOG00000022196
10923799	Cd28	CD28 antigen (Cd28), mRNA.
10766123	Cdc42bpa	CDC42 binding protein kinase alpha (Cdc42bpa), mRNA.
10837114	Cdk105|LOC317618	CDK105 protein (Cdk105), mRNA.
10820688	Cdk105|LOC681386	CDK105 protein (Cdk105), mRNA.
10826002	Celsr2	similar to Cadherin EGF LAG seven-pass G-type receptor 2 precursor gene:ENSRNOG00000020058
10913272	Celsr3	cadherin EGF LAG seven-pass G-type receptor 3 (Celsr3), mRNA.
10712853	Clcf1	cardiotrophin-like cytokine factor 1 (Clcf1), mRNA.
10729024	Cntf	ciliary neurotrophic factor (Cntf), mRNA.
10882367	Cntfr	ciliary neurotrophic factor receptor (Cntfr), mRNA.
10736033	Crk	v-crk sarcoma virus CT10 oncogene homolog (avian) (Crk), mRNA.
10797588	Ctsg	30 kDa protein gene:ENSRNOG00000020647
10799745	Cubn	cubilin (intrinsic factor-cobalamin receptor) (Cubn), mRNA.
10769695	Ddr2	discoidin domain receptor family, member 2 (Ddr2), mRNA.
10919716	Dnajc13	DnaJ (Hsp40) homolog, subfamily C, member 13 (Dnajc13), mRNA.
10844302	Dnm1	dynamin 1 (Dnm1), mRNA.
10753393	Dscam	Down syndrome cell adhesion molecule (Dscam), mRNA.
10753419	Dscam	Down syndrome cell adhesion molecule (Dscam), mRNA.
10915555	Edg8	endothelial differentiation, sphingolipid G-protein-coupled receptor, 8 (Edg8), mRNA.
10826725	Egf	epidermal growth factor (Egf), mRNA.
10774274	Egfr	epidermal growth factor receptor (Egfr), mRNA.
10719204	Ehd2	EH-domain containing 2 (Ehd2), mRNA.
10888596	Ehd3	EH-domain containing 3 (Ehd3), mRNA.
10848733	Ehd4	EH-domain containing 4 (Ehd4), mRNA.
10931222	Emr1	EGF-like module containing, mucin-like, hormone receptor-like sequence 1 (Emr1), mRNA.
10873706	Epha2	Eph receptor A2 (Epha2), mRNA.
10880738	Epha8	Ephrin type-A receptor 8 gene:ENSRNOG00000013036
10870902	Eps15	epidermal growth factor receptor pathway substrate 15 (Eps15), mRNA.
10787162	Eps15l1	epidermal growth factor receptor pathway substrate 15-like 1 (Eps15l1), mRNA.
10866544	Eps8	similar to epidermal growth factor receptor pathway substrate 8 gene:ENSRNOG00000007047
10737962	Erbb2	v-erb-b2 erythroblastic leukemia viral oncogene homolog 2, neuro/glioblastoma derived oncogene homolog (avian) (Erbb2), mRNA.
10899839	Erbb3	v-erb-b2 erythroblastic leukemia viral oncogene homolog 3 (avian) (Erbb3), mRNA.
10928636	Erbb4	v-erb-a erythroblastic leukemia viral oncogene homolog 4 (avian) (Erbb4), mRNA.
10775873	Ereg	epiregulin (Ereg), mRNA.
10820586	F2r	coagulation factor II (thrombin) receptor (F2r), mRNA.
10812589	F2rl2	coagulation factor II (thrombin) receptor-like 2 (F2rl2), mRNA.
10812331	Faim	Fas apoptotic inhibitory molecule (Faim), mRNA.
10912456	Faim	Fas apoptotic inhibitory molecule (Faim), mRNA.
10714890	Fas	Fas (TNF receptor superfamily, member 6) (Fas), mRNA.
10813172	Fgf10	fibroblast growth factor 10 (Fgf10), mRNA.
10900789	Fgf22	fibroblast growth factor 22 (Fgf22), mRNA.
10712618	Fgf3	fibroblast growth factor 3 (Fgf3), mRNA.
10775586	Fgf5	fibroblast growth factor 5 (Fgf5), mRNA.
10858999	Fgf6	fibroblast growth factor 6 (Fgf6), mRNA.
10839434	Fgf7	fibroblast growth factor 7 (Fgf7), mRNA.
10780813	Fgf9	fibroblast growth factor 9 (Fgf9), mRNA.
10773146	Fgfbp1	fibroblast growth factor binding protein 1 (Fgfbp1), mRNA.
10792304	Fgfr1	Fibroblast growth factor receptor 1 (Fgfr1), mRNA.
10726172	Fgfr2	fibroblast growth factor receptor 2 (Fgfr2), transcript variant a, mRNA.
10777748	Fgfr3	fibroblast growth factor receptor 3 (Fgfr3), mRNA.

10797323	Fgfr4	fibroblast growth factor receptor 4 (Fgfr4), mRNA.
10774992	Fgfrl1	fibroblast growth factor receptor-like 1 (Fgfrl1), mRNA.
10937725	Figf	c-fos induced growth factor (Figf), mRNA.
10756418	Flt1	FMS-like tyrosine kinase 1 (Flt1), mRNA.
10733102	Flt4	FMS-like tyrosine kinase 4 (Flt4), mRNA.
10833814	Foxo3a	forkhead box O3a (Foxo3a), mRNA.
10709336	Frag1	FGF receptor activating protein 1 (Frag1), mRNA.
10902524	Frs2	fibroblast growth factor receptor substrate 2 (Frs2), mRNA.
10926310	Frs3	fibroblast growth factor receptor substrate 3 (Frs3), mRNA.
10885464	Fut8	fucosyltransferase 8 (Fut8), mRNA.
10853469	Fzd1	frizzled homolog 1 (Drosophila) (Fzd1), mRNA.
10738591	Fzd2	frizzled homolog 2 (Drosophila) (Fzd2), mRNA.
10806927	Gab1	growth factor receptor bound protein 2-associated protein 1 (Gab1), mRNA.
10708745	Gab2	growth factor receptor bound protein 2-associated protein 2 (Gab2), mRNA.
10789442	Gas6	growth arrest specific 6 (Gas6), mRNA.
10857036	Gfpt1	glutamine fructose-6-phosphate transaminase 1 (Gfpt1), mRNA.
10731075	Gfra1	glial cell line derived neurotrophic factor family receptor alpha 1 (Gfra1), mRNA.
10781410	Gfra2	glial cell line derived neurotrophic factor family receptor alpha 2 (Gfra2), mRNA.
10803815	Gfra3	glial cell line derived neurotrophic factor family receptor alpha 3 (Gfra3), mRNA.
10850004	Gfra4	glial cell line derived neurotrophic factor family receptor alpha 4 (Gfra4), mRNA.
10778505	Grb10	growth factor receptor bound protein 10 (Grb10), mRNA.
10845751	Grb14	growth factor receptor bound protein 14 (Grb14), mRNA.
10721229	Grb2	mRNA for Ash-psi, complete cds.
10749005	Grb2	growth factor receptor bound protein 2 (Grb2), mRNA.
10737999	Grb7	growth factor receptor bound protein 7 (Grb7), mRNA.
10803947	Hbegf	heparin-binding EGF-like growth factor (Hbegf), mRNA.
10830303	Hdac2	similar to Histone deacetylase 2 gene:ENSRNOG00000000604
10742128	Hmmr	hyaluronan mediated motility receptor (RHAMM) (Hmmr), mRNA.
10894695	Igf1	insulin-like growth factor 1 (Igf1), transcript variant 3, mRNA.
10707889	Igf1r	insulin-like growth factor 1 receptor (Igf1r), mRNA.
10726999	Igf2	insulin-like growth factor 2 (Igf2), mRNA.
10702996	Igf2r	insulin-like growth factor 2 receptor (Igf2r), mRNA.
10939668	Igsf1	immunoglobulin superfamily, member 1 (Igsf1), mRNA.
10938291	Il1rapl1	interleukin 1 receptor accessory protein-like 1 (Il1rapl1), mRNA.
10922857	Il1rl1	interleukin 1 receptor-like 1 (Il1rl1), mRNA.
10813007	Il6st	interleukin 6 signal transducer (Il6st), mRNA.
10907689	Itga5	integrin alpha 5 (Itga5), mRNA.
10845587	Itgb6	integrin, beta 6 (Itgb6), mRNA.
10889036	Kcnk3	potassium channel, subfamily K, member 3 (Kcnk3), mRNA.
10883927	Kidins220	kinase D-interacting substrate of 220 kDa (Kidins220), mRNA.
10785531	Klhl1	kelch-like 1 (Drosophila) (Klhl1), mRNA.
10859164	Klrd1|Klre1	killer cell lectin-like receptor, subfamily D, member 1 (Klrd1), mRNA.
10773853	Lif	leukemia inhibitory factor (Lif), mRNA.
10813445	Lifr	leukemia inhibitory factor receptor (Lifr), mRNA.
10917711	Lingo1	similar to leucine-rich repeat neuronal 6A isoform 1 gene:ENSRNOG00000017193
10938446	Maged1	melanoma antigen, family D, 1 (Maged1), mRNA.
10937492	Mageh1	melanoma antigen, family H, 1 (Mageh1), mRNA.
10853819	2|Asz1|Cav1|Cttnbp2|S	met proto-oncogene (Met), mRNA.
10913155	Mst1	Macrophage stimulating 1 (hepatocyte growth factor-like) (Mst1), mRNA.
10913043	Mst1r	macrophage stimulating 1 receptor (c-met-related tyrosine kinase) (Mst1r), mRNA.
10915124	Mtnr1b	similar to melatonin receptor 1B gene:ENSRNOG00000008972
10754735	Muc4	Fisher 344 pre-sialomucin complex (pSMC) mRNA, repeat sequences 10-14, partial cds.
10917183	Ncam1	neural cell adhesion molecule 1 (Ncam1), mRNA.
10707649	Ndn	necdin (Ndn), mRNA.
10879427	Nfyc	nuclear transcription factor-Y gamma (Nfyc), mRNA.
10929660	Ngef	brain-enriched SH3-domain protein mRNA, complete cds.
10817894	Ngfb	similar to nerve growth factor, beta gene:ENSRNOG00000016571
10746538	Ngfr	nerve growth factor receptor (TNFR superfamily, member 16) (Ngfr), mRNA.
10935047	Ngfrap1	nerve growth factor receptor (TNFRSF16) associated protein 1 (Ngfrap1), mRNA.
10843793	Notch1	Notch gene homolog 1 (Drosophila) (Notch1), mRNA.
10900907	Notch3	Notch gene homolog 3 (Drosophila) (Notch3), mRNA.
10933924	Nr0b1	nuclear receptor subfamily 0, group B, member 1 (Nr0b1), mRNA.
10899387	Nr4a1	nuclear receptor subfamily 4, group A, member 1 (Nr4a1), mRNA.
10845384	Nr4a2	nuclear receptor subfamily 4, group A, member 2 (Nr4a2), mRNA.
10844858	Nr5a1	Steroidogenic factor 1 gene:ENSRNOG00000012682
10844869	Nr6a1	Germ cell nuclear factor gene:ENSRNOG00000013232
10920461	Nradd	neurotrophin receptor associated death domain (Nradd), mRNA.
10788627	Nrg1	neuregulin 1 (Nrg1), mRNA.
10803929	Nrg2	Isoform 2 of Pro-neuregulin-2, membrane-bound isoform precursor gene:ENSRNOG00000019093
10930890	Nrtn	neurturin (Nrtn), mRNA.
10706807	Ntf5	neurotrophin 5 (Ntf5), mRNA.
10824188	Ntrk1	neurotrophic tyrosine kinase, receptor, type 1 (Ntrk1), mRNA.
10797089	Ntrk2	neurotrophic tyrosine kinase, receptor, type 2 (Ntrk2), mRNA.
10722864	Ntrk3	neurotrophic tyrosine kinase, receptor, type 3 (Ntrk3), mRNA.
10842839	Ogfr	opioid growth factor receptor (Ogfr), mRNA.
10927041	Ogfrl1	opioid growth factor receptor-like 1 (Ogfrl1), mRNA.
10821698	Osmr	oncostatin M receptor (Osmr), mRNA.
10816026	Pdgfc	platelet-derived growth factor, C polypeptide (Pdgfc), mRNA.
10776459	Pdgfra	platelet derived growth factor receptor, alpha polypeptide (Pdgfra), mRNA.
10802040	Pdgfrb	platelet derived growth factor receptor, beta polypeptide (Pdgfrb), mRNA.
10919453	Pik3cb	phosphatidylinositol 3-kinase, catalytic, beta polypeptide (Pik3cb), mRNA.
10841850	Plcg1	phospholipase C, gamma 1 (Plcg1), mRNA.
10754179	Popdc2	popeye domain containing 2 (Popdc2), mRNA.
10807019	Pou4f2	similar to POU domain, class 4, transcription factor 2 gene:ENSRNOG00000012167

10857984	Pparg	peroxisome proliferator activated receptor gamma (Pparg), mRNA.
10777422	Ppp2r2c	protein phosphatase 2 (formerly 2A), regulatory subunit B (PR 52), gamma isoform (Ppp2r2c), mRNA.
10894512	Prdm4	PR domain containing 4 (Prdm4), mRNA.
10889789	Prkd1	Serine/threonine-protein kinase D1 gene:ENSRNOG00000004165
10863820	Prokr1	prokineticin receptor 1 (Prokr1), mRNA.
10850137	Prokr2	prokineticin receptor 2 (Prokr2), mRNA.
10842475	Ptpn1	protein tyrosine phosphatase, non-receptor type 1 (Ptpn1), mRNA.
10782609	Ptprg	protein tyrosine phosphatase, receptor type, G (Ptprg), mRNA.
10717118	Ptprk	protein tyrosine phosphatase, receptor type, K, extracellular region (Ptprk), mRNA.
10864918	Ret	ret proto-oncogene (Ret), transcript variant 1, mRNA.
10741544	Rhbdl1	similar to rhomboid, veinlet-like 1 gene:ENSRNOG00000019921
10726650	d2|RGD1559566|RGD	ribosomal protein L9 (Rpl9), mRNA.
10932126	d2|RGD1559566|RGD	ribosomal protein L9 (Rpl9), mRNA.
10822520	)1559963|RGD156178	60S ribosomal protein L9 gene:ENSRNOG00000026716
10776256	:pl9|Ehd2|RGD156178	ribosomal protein L9 (Rpl9), mRNA.
10719210	d2|RGD1561789|RGD	ribosomal protein L9 (Rpl9), mRNA.
10754136	d2|RGD1561789|RGD	ribosomal protein L9 (Rpl9), mRNA.
10772718	d2|RGD1561789|RGD	ribosomal protein L9 (Rpl9), mRNA.
10834475	d2|RGD1561789|RGD	ribosomal protein L9 (Rpl9), mRNA.
10873870	d2|RGD1561789|RGD	ribosomal protein L9 (Rpl9), mRNA.
10901711	d2|RGD1561789|RGD	ribosomal protein L9 (Rpl9), mRNA.
10846872	Rtn4rl2	reticulon 4 receptor-like 2 (Rtn4rl2), mRNA.
10794242	Sema4d	similar to semaphorin 4D gene:ENSRNOG00000013679
10757545	Sh2b2	SH2B adaptor protein 2 (Sh2b2), mRNA.
10869728	Sh3gl2	similar to SH3-domain GRB2-like 2 gene:ENSRNOG00000006761
10708338	Sh3gl3	SH3-domain GRB2-like 3 (Sh3gl3), mRNA.
10816791	Shc1	src homology 2 domain-containing transforming protein C1 (Shc1), mRNA.
10794261	Shc3	src homology 2 domain-containing transforming protein C3 (Shc3), mRNA.
10794812	Slc22a23	similar to ion transporter protein gene:ENSRNOG00000017210
10918326	Snx1	sorting nexin 1 (Snx1), mRNA.
10850390	Snx5	sorting nexin 5 (Snx5), mRNA.
10882773	Socs5	suppressor of cytokine signaling 5 (Socs5), mRNA.
10784980	Sorbs3	sorbin and SH3 domain containing 3 (Sorbs3), mRNA.
10888021	Sos1	Son of sevenless homolog 1 (Drosophila) (Sos1), mRNA.
10803953	Sra1	steroid receptor RNA activator 1 (Sra1), mRNA.
10717256	Stx7	syntaxin 7 (Stx7), mRNA.
10753075	Synj1	145 kDa synaptojanin isoform mRNA, complete cds.
10832124	Tff3	trefoil factor 3, intestinal (Tff3), mRNA.
10856948	Tgfa	transforming growth factor alpha (Tgfa), mRNA.
10868923	Tgfbr1	transforming growth factor, beta receptor 1 (Tgfbr1), mRNA.
10920745	Tgfbr2	transforming growth factor, beta receptor II (Tgfbr2), mRNA.
10771070	Tgfbr3	transforming growth factor, beta receptor III (Tgfbr3), mRNA.
10927680	Tgfbrap1	transforming growth factor, beta receptor associated protein 1 (Tgfbrap1), mRNA.
10781321	Tnfrsf10b	tumor necrosis factor receptor superfamily, member 10b (Tnfrsf10b), mRNA.
10858967	Tnfrsf1a	tumor necrosis factor receptor superfamily, member 1a (Tnfrsf1a), mRNA.
10874829	Tnfrsf4	tumor necrosis factor receptor superfamily, member 4 (Tnfrsf4), mRNA.
10744171	Tnfsf12	tumor necrosis factor ligand superfamily member 12 (Tnfsf12), mRNA.
10882050	Trp73	transformation related protein 73 (Trp73), mRNA.
10838881	Tyro3	TYRO3 protein tyrosine kinase 3 (Tyro3), mRNA.
10797351	Unc5a	unc-5 homolog A (C. elegans) (Unc5a), mRNA.
10833013	Unc5b	unc-5 homolog B (C. elegans) (Unc5b), mRNA.
10791565	Vegfc	vascular endothelial growth factor C (Vegfc), mRNA.
10714576	Vldlr	very low density lipoprotein receptor (Vldlr), mRNA.
10708908	Wnt11	similar to Protein Wnt-11 precursor gene:ENSRNOG00000015982
10917116	Zbtb16	zinc finger and BTB domain containing 16 (Zbtb16), mRNA.
