# Supplementary material for: Intrinsic response of thoracic propriospinal neurons to axotomy
Source: BMC Neurosci. 2010 Jun 4;11:69. doi: 10.1186/1471-2202-11-69 (PMC2894843; doi:10.1186/1471-2202-11-69)
Supplement: Additional file 10 — Complete list of genes compiled for the apoptosis and cell death gene programs. [file 1471-2202-11-69-S10.PDF]

Additional File 10

Pro and Anti Apoptotic Genes

Probe ID	Gene Symbol	Gene Title
10745731	Aatf	apoptosis antagonizing transcription factor (Aatf), mRNA.
10816834	Adar	adenosine deaminase, RNA-specific (Adar), mRNA.
10804774	Adrb2	adrenergic receptor, beta 2 (Adrb2), mRNA.
10939595	Aifm1	apoptosis-inducing factor, mitochondrion-associated 1 (Aifm1), nuclear gene encoding mitochondrial protein, mRNA.
10830063	Aifm2	apoptosis-inducing factor, mitochondrion-associated 2 (Aifm2), nuclear gene encoding mitochondrial protein, mRNA.
10770197	Akt3	thymoma viral proto-oncogene 3 (Akt3), mRNA.
10883025	Alk	Anaplastic lymphoma kinase gene:ENSRNOG00000008683
10900405	Amh	anti-Mullerian hormone (Amh), mRNA.
10901783	Apaf1	apoptotic peptidase activating factor 1 (Apaf1), mRNA.
10770048	Apcs	serum amyloid P-component (Apcs), mRNA.
10817446	Aph1a	anterior pharynx defective 1a homolog (C. elegans) (Aph1a), mRNA.
10847693	Api5	cDNA clone MGC:187857 IMAGE:9089120, complete cds.
10904511	Arc	activity regulated cytoskeletal-associated protein (Arc), mRNA.
10749675	Arhgdia	Rho GDP dissociation inhibitor (GDI) alpha (Arhgdia), mRNA.
10817396	Arnt	aryl hydrocarbon receptor nuclear translocator (Arnt), mRNA.
10857950	Atg7	autophagy related 7 homolog (S. cerevisiae) (Atg7), mRNA.
10929086	Atg9a	autophagy-related 9A (yeast) (Atg9a), mRNA.
10852920	Atg9b	similar to nitric oxide synthase 3 antisense gene:ENSRNOG00000023449
10917385	Atm	ataxia telangiectasia mutated homolog (human) (Atm), mRNA.
10898335	Atxn10	ataxin 10 (Atxn10), mRNA.
10838512	Aven	apoptosis, caspase activation inhibitor (Aven), mRNA.
10713458	Bad	bcl2-associated death promoter (Bad), mRNA.
10876052	Bag1	Bcl2-associated athanogene 1 (Bag1), mRNA.
10831816	Bak1	BCL2-antagonist/killer 1 (Bak1), mRNA.
10928684	Bard1	BRCA1 associated RING domain 1 (Bard1), mRNA.
10721834	Bax	Apoptosis regulator BAX gene:ENSRNOG00000020876
10738460	Bax	Bcl2-associated X protein (Bax), mRNA.
10917954	Bbs4	Bardet-Biedl syndrome 4 homolog (human) (Bbs4), mRNA.
10819653	Bcl10	B-cell leukemia/lymphoma 10 (Bcl10), mRNA.
10766953	Bcl2	B-cell leukemia/lymphoma 2 (Bcl2), mRNA.
10912112	Bcl2a1	B-cell leukemia/lymphoma 2 related protein A1 (Bcl2a1), mRNA.
10850826	Bcl2l1	Bcl2-like 1 (Bcl2l1), nuclear gene encoding mitochondrial protein, transcript variant 2, mRNA.
10724228	|LOC293190|LOC	bcl-x short form mRNA, complete cds.
10911690	Bcl2l10	Bcl2-like 10 (Bcl2l10), mRNA.
10858362	Bcl2l13	BCL2-like 13 (apoptosis facilitator) (Bcl2l13), mRNA.
10859237	Bcl2l14	Bcl2-like 14 (apoptosis facilitator) (Bcl2l14), mRNA.
10780239	Bcl2l2|Pabpn1	Bcl2-like 2 (Bcl2l2), mRNA.
10747620	Becn1	beclin 1, autophagy related (Becn1), transcript variant 1, mRNA.
10731229	Bfar	bifunctional apoptosis regulator (Bfar), mRNA.
10935025	Bhlhb9	basic helix-loop-helix domain containing, class B, 9 (Bhlhb9), mRNA.
10865222	Bid	BH3 interacting domain death agonist (Bid), mRNA.
10905822	Bik	Bcl2-interacting killer (Bik), mRNA.
10914788	Birc2	baculoviral IAP repeat-containing 2 (Birc2), mRNA.
10914799	Birc3	baculoviral IAP repeat-containing 3 (Birc3), mRNA.
10921195	Birc4	clone 2 inhibitor of apoptosis protein 3 mRNA, complete cds.
10936198	Birc4	baculoviral IAP repeat-containing 4 (Birc4), mRNA.
10848444	Bmf	Bcl2 modifying factor (Bmf), mRNA.
10923687	Bmpr2	similar to Bone morphogenetic protein receptor type-2 precursor gene:ENSRNOG00000022196
10726457	Bnip3	BCL2/adenovirus E1B 19 kDa-interacting protein 3 (Bnip3), nuclear gene encoding mitochondrial protein, mRNA.
10927826	Bnip3	BCL2/adenovirus E1B 19 kDa-interacting protein 3 (Bnip3), nuclear gene encoding mitochondrial protein, mRNA.
10784694	Bnip3l	BCL2/adenovirus E1B interacting protein 3-like (Bnip3l), mRNA.
10925636	Bok	Bcl-2-related ovarian killer protein (Bok), mRNA.
10806677	Btbd14b	BTB (POZ) domain containing 14B (Btbd14b), mRNA.
10895069	Btg1	B-cell translocation gene 1, anti-proliferative (Btg1), mRNA.
10880727	C1qb	complement component 1, q subcomponent, beta polypeptide (C1qb), mRNA.
10865369	C3ar1	complement component 3a receptor 1 (C3ar1), mRNA.
10767436	C4bpb	complement component 4 binding protein, beta (C4bpb), mRNA.
10821666	C7	similar to complement component 7 precursor gene:ENSRNOG00000012516
10770313	Cabc1|Psen2	chaperone, ABC1 activity of bc1 complex like (S. pombe) (Cabc1), nuclear gene encoding mitochondrial protein, mRNA.
10843697	Card9	caspase recruitment domain family, member 9 (Card9), mRNA.
10907815	Casp1	caspase 1 (Casp1), mRNA.
10907834	Casp12	caspase 12 (Casp12), mRNA.
10894140	Casp14	similar to Caspase-14 precursor gene:ENSRNOG00000007352
10855020	Casp2	caspase 2 (Casp2), mRNA.
10791652	Casp3	caspase 3, apoptosis related cysteine protease (Casp3), mRNA.
10716226	Casp7	caspase 7 (Casp7), mRNA.
10923595	Casp8	caspase 8 (Casp8), mRNA.
10873768	Casp9	caspase 9 (Casp9), mRNA.
10916669	Cbl	similar to Casitas B-lineage lymphoma gene:ENSRNOG00000008444
10833239	Ccar1	cell division cycle and apoptosis regulator 1 (Ccar1), mRNA.
10805313	Ccdc5	coiled-coil domain containing 5 (Ccdc5), mRNA.
10923799	Cd28	CD28 antigen (Cd28), mRNA.
10802013	Cd74	CD74 antigen (invariant polypeptide of major histocompatibility complex, class II antigen-associated) (Cd74), mRNA.
10913553	Cdc25a	cell division cycle 25 homolog A (S. pombe) (Cdc25a), mRNA.
10851724	Cdh22	cadherin 22 (Cdh22), mRNA.
10844355	Cdk9	cyclin-dependent kinase 9 (CDC2-related kinase) (Cdk9), mRNA.
10727056	Cdkn1c	cyclin-dependent kinase inhibitor 1C (P57) (Cdkn1c), transcript variant 1, mRNA.
10713074	Cfl1	cofilin 1, non-muscle (Cfl1), mRNA.
10923580	Cflar	CASP8 and FADD-like apoptosis regulator (Cflar), transcript variant 2, mRNA.
10838831	Chp|RGD1565588	calcium binding protein p22 (Chp), mRNA.
10859965	Chp|RGD1565588	calcium binding protein p22 (Chp), mRNA.
10876827	Chp|RGD1565588	calcium binding protein p22 (Chp), mRNA.
10805986	Ciapin1	cytokine induced apoptosis inhibitor 1 (Ciapin1), mRNA.
10775862	Cipar1	castration induced prostatic apoptosis-related protein 1 (Cipar1), mRNA.
10762537	Cit	citron (rho-interacting, serine/threonine kinase 21) (Cit), mRNA.
10712853	Clcf1	cardiotrophin-like cytokine factor 1 (Clcf1), mRNA.
10822631	Cldn11	claudin 11 (Cldn11), mRNA.
10831090	Skiv2l|Ehmt2|Stk19	chloride intracellular channel 1 (Clic1), mRNA.
10829378	Col18a1	similar to Collagen alpha-1(XVIII) chain precursor gene:ENSRNOG00000001229
10743186	Cops3	COP9 (constitutive photomorphogenic) homolog, subunit 3 (Arabidopsis thaliana) (Cops3), mRNA.
10901993	Cradd	CASP2 and RIPK1 domain containing adaptor with death domain (Cradd), mRNA.
10836490	Csnrp3	similar to TGF-beta induced apoptosis protein 2 gene:ENSRNOG00000005359
10797588	Ctsg	30 kDa protein gene:ENSRNOG00000020647
10796234	Cugbp2	CUG triplet repeat, RNA binding protein 2 (Cugbp2), transcript variant 1, mRNA.
10744460	Cxcl16	chemokine (C-X-C motif) ligand 16 (Cxcl16), mRNA.
10783529	Dad1	defender against cell death 1 (Dad1), mRNA.
10814098	Dap	death-associated protein (Dap), mRNA.
10797032	Dapk1	death associated protein kinase 1 (Dapk1), mRNA.
10900296	Dapk3	death-associated protein kinase 3 (Dapk3), mRNA.
10877573	Dbc1	deleted in bladder cancer 1 (Dbc1), mRNA.
10895861	Ddit3	DNA-damage inducible transcript 3 (Ddit3), transcript variant 1, mRNA.
10832920	Ddit4	DNA-damage-inducible transcript 4 (Ddit4), mRNA.
10811041	Ddx19	DEAD (Asp-Glu-Ala-Asp) box polypeptide 19 (Ddx19), mRNA.

10924507	Des	desmin (Des), mRNA.
10874070	Dffa	DNA fragmentation factor, alpha subunit (Dffa), mRNA.
10882025	Dffb	DNA fragmentation factor, beta subunit (Dffb), mRNA.
10758432	Diablo	diablo homolog (Drosophila) (Diablo), nuclear gene encoding mitochondrial protein, mRNA.
10872321	Diablo	diablo homolog (Drosophila) (Diablo), nuclear gene encoding mitochondrial protein, mRNA.
10723971	Dnajb13	DnaJ (Hsp40) related, subfamily B, member 13 (Dnajb13), mRNA.
10889766	Dnajb9	DnaJ (Hsp40) homolog, subfamily B, member 9 (Dnajb9), mRNA.
10740713	Dnase1	deoxyribonuclease I (Dnase1), mRNA.
10779496	Dnase1l3	deoxyribonuclease 1-like 3 (Dnase1l3), mRNA.
10758986	Dynll1	dynein light chain LC8-type 1 (Dynll1), mRNA.
10751396	Eaf2	ELL associated factor 2 (Eaf2), mRNA.
10804805	Ecg2	Serine protease inhibitor Kazal-type 7 precursor gene:ENSRNOG00000032873
10835150	Endog	endonuclease G (Endog), nuclear gene encoding mitochondrial protein, mRNA.
10729610	Ermp1	endoplasmic reticulum metallopeptidase 1 (Ermp1), mRNA.
10814705	Evi1	ecotropic viral integration site 1 (Evi1), mRNA.
10822611	Evi1	ecotropic viral integration site 1 (Evi1), mRNA.
10813934	Zfp622|Fam134b	Zinc finger protein 622 gene:ENSRNOG00000010589
10714890	Fas	Fas (TNF receptor superfamily, member 6) (Fas), mRNA.
10769231	Fasl	Fas ligand (TNF superfamily, member 6) (Fasl), mRNA.
10712618	Fgf3	fibroblast growth factor 3 (Fgf3), mRNA.
10773146	Fgfbp1	fibroblast growth factor binding protein 1 (Fgfbp1), mRNA.
10927692	Fhl2	four and a half LIM domains 2 (Fhl2), mRNA.
10757688	Fkbp6	FK506 binding protein 6 (Fkbp6), mRNA.
10854139	Flnc	similar to Filamin-C (Gamma-filamin) (Filamin-2) (Protein FLNc) (Actin-binding-like protein) (ABP-L) (ABP-280-like protein) isoform 2 gene:ENSRNOG00000007281
10900358	Gadd45b	growth arrest and DNA-damage-inducible 45 beta (Gadd45b), mRNA.
10855387	Gimap4	GTPase, IMAP family member 4 (Gimap4), mRNA.
10812216	Glrx1	glutaredoxin 1 (thioltransferase) (Glrx1), mRNA.
10920956	Gorasp1	golgi reassembly stacking protein 1 (Gorasp1), mRNA.
10848281	Grem1	gremlin 1 (Grem1), mRNA.
10835757	Gsn	gelsolin (Gsn), mRNA.
10731428	Gspt1	G1 to S phase transition 1 (Gspt1), mRNA.
10784049	Gzmb	R.norvegicus mRNA for granzyme-like protein III.
10784054	RGD1561819|Gzm	similar to Natural killer cell protease 1 precursor gene:ENSRNOG00000039015
10819322	H2afz	H2A histone family, member Z (H2afz), mRNA.
10749070	H3f3b	H3 histone, family 3B (H3f3b), mRNA.
10753921	H3f3b	H3 histone, family 3B, mRNA (cDNA clone MGC:187497 IMAGE:7386252), complete cds.
10840975	Hck	hemopoietic cell kinase (Hck), mRNA.
10880012	Hdac1	histone deacetylase 1 (Hdac1), mRNA.
10929261	Hdac1	histone deacetylase 1 (Hdac1), mRNA.
10747752	Hdac5	similar to Histone deacetylase 5 gene:ENSRNOG00000020905
10886786	Hdmcp	mitochondrial hepatocellular carcinoma-downregulated carrier protein (Hdmcp), nuclear gene encoding mitochondrial protein, mRNA.
10914415	Higd1a	HIG1 domain family, member 1A (Higd1a), mRNA.
10798455	|LOC684841|LOC	histone cluster 1, H2ai (Hist1h2ai), mRNA.
10798463	511|Hist1h3g|LOC	Histone H3.1 gene:ENSRNOG00000038900
10798505	511|Hist1h3g|LOC	Histone H3.1 gene:ENSRNOG00000038829
10828575	Hmga1	high mobility group AT-hook 1 (Hmga1), mRNA.
10737705	Hoxb13	homeo box B13 (Hoxb13), mRNA.
10899603	Hoxc9	similar to Homeobox protein Hox-C9 gene:ENSRNOG00000028619
10899599	Hoxc6|Hoxc9	similar to Homeobox protein Hox-C9 (Hox-3.2) isoform 2 gene:ENSRNOG00000016199
10762426	Hrk	harakiri, BCL2 interacting protein (contains only BH3 domain) (Hrk), mRNA.
10909482	Hyou1	hypoxia up-regulated 1 (Hyou1), transcript variant 1, mRNA.
10789087	Ikbkb	inhibitor of kappaB kinase beta (Ikbkb), mRNA.
10703953	Il11	interleukin 11 (Il11), mRNA.
10859799	Il6	interleukin 6 (Il6), mRNA.
10709629	Ilk	integrin linked kinase (Ilk), mRNA.
10903053	Inhbc	inhibin beta C (Inhbc), mRNA.
10898862	Irak4	interleukin-1 receptor-associated kinase 4 (Irak4), mRNA.
10733553	Irf1	interferon regulatory factor 1 (Irf1), mRNA.
10785244	Itm2b	integral membrane protein 2B (Itm2b), mRNA.
10878112	Jun	Jun oncogene (Jun), mRNA.
10903459	Klf10	Kruppel-like factor 10 (Klf10), mRNA.
10790670	Klf2	Kruppel-like factor 2 (lung) (Klf2), mRNA.
10860691	Krit1	KRIT1, ankyrin repeat containing (Krit1), mRNA.
10872489	Laptm5	lysosomal-associated protein transmembrane 5 (Laptm5), mRNA.
10880033	Lck	cDNA clone MGC:188286 IMAGE:7381544, complete cds.
10844331	Lcn2	lipocalin 2 (Lcn2), mRNA.
10757768	Limk1	LIM domain kinase 1 (Limk1), mRNA.
10819562	Lmo4	LIM domain only 4 (Lmo4), mRNA.
10862178	LOC286911	cationic trypsinogen (LOC286911), mRNA.
10888617	LOC298795	similar to 14-3-3 protein sigma, mRNA (cDNA clone MGC:108952 IMAGE:7112967), complete cds.
10755196	LOC303823	similar to mitogen-activated protein kinase kinase kinase 13; leucine zipper-bearing kinase, mRNA (cDNA clone MGC:94134 IMAGE:7127060), complete cds.
10815226	C689629|LOC499	similar to GTPase activating protein testicular GAP1 gene:ENSRNOG00000023039
10816613	LOC499653	Dingo protein (LOC499653), mRNA.
10842121	C499941|RGD1311	SCF apoptosis response protein 1 (LOC499941), mRNA.
10914823	Yap1|LOC654482	Neuron-specific YAPdeltaC insert61 isoform gene:ENSRNOG00000005933
10937594	Pik3ca|LOC68559	125 kDa protein gene:ENSRNOG00000010458
10812354	C309957|LOC685	Myocyte-specific enhancer factor 2A gene:ENSRNOG00000032789
10789164	Nek3|LOC686318	PREDICTED: NIMA (never in mitosis gene a)-related expressed kinase 3 (Nek3), mRNA.
10902409	LOC688019	similar to THAP domain containing, apoptosis associated protein 2 gene:ENSRNOG00000003993
10749533	LOC688299|Aatk	similar to apoptosis-associated tyrosine kinase gene:ENSRNOG00000004392
10795986	LOC690057	similar to modulator of apoptosis 1 gene:ENSRNOG00000033970
10721028	Lsm14a	similar to Cytokinesis, Apoptosis, RNA-associated family member gene:ENSRNOG00000021133
10828016	Lta	lymphotoxin A (Lta), mRNA.
10913817	Ltf	lactotransferrin (Ltf), mRNA.
10715644	Lzts2	leucine zipper, putative tumor suppressor 2 (Lzts2), mRNA.
10847308	Madd	MAP-kinase activating death domain (Madd), mRNA.
10938446	Maged1	melanoma antigen, family D, 1 (Maged1), mRNA.
10743668	Map2k4	mitogen activated protein kinase kinase 4 (Map2k4), mRNA.
10821276	Map3k1	mitogen activated protein kinase kinase kinase 1 (Map3k1), mRNA.
10720111	Map3k10	similar to mitogen-activated protein kinase kinase kinase 10 gene:ENSRNOG00000023521
10713102	Map3k11	mitogen-activated protein kinase kinase kinase 11 (Map3k11), mRNA.
10890991	Map3k9	similar to mitogen-activated protein kinase kinase kinase 9 gene:ENSRNOG00000007271
10771267	Mapk10	mitogen activated protein kinase 10 (Mapk10), mRNA.
10847509	Mapk8ip1	mitogen-activated protein kinase 8 interacting protein 1 (Mapk8ip1), mRNA.
10752650	MGC95208	similar to 4930453N24Rik protein (MGC95208), mRNA.
10933015	MGC95208	similar to 4930453N24Rik protein (MGC95208), mRNA.
10891861	Moap1	modulator of apoptosis 1 (Moap1), mRNA.
10784517	Msra	methionine sulfoxide reductase A (Msra), mRNA.
10913155	Mst1	Macrophage stimulating 1 (hepatocyte growth factor-like) (Mst1), mRNA.
10726593	Msx3	Homeo box, msh-like 3 gene:ENSRNOG00000018324
10837728	Mtch2	mitochondrial carrier homolog 2 (C. elegans) (Mtch2), nuclear gene encoding mitochondrial protein, mRNA.
10778038	Mtp18	mitochondrial protein 18 kDa (Mtp18), nuclear gene encoding mitochondrial protein, mRNA.
10816749	Muc1	similar to mucin 1, transmembrane gene:ENSRNOG00000020539
10896814	Myc	myelocytomatosis oncogene (Myc), mRNA.
10937225	Mycs	myc-like oncogene, s-myc protein (Mycs), mRNA.
10721865	Myd116	myeloid differentiation primary response gene 116 (Myd116), mRNA.
10920860	Myd88	myeloid differentiation primary response gene 88 (Myd88), mRNA.

10820908	RGD1559914|Naip	similar to baculoviral IAP repeat-containing 1e gene:ENSRNOG00000033693
10718725	Nalp5	NACHT, leucine rich repeat and PYD containing 5 (Nalp5), mRNA.
10904169	Ndrg1	N-myc downstream regulated gene 1 (Ndrg1), mRNA.
10862698	Neurod6	neurogenic differentiation 6 (Neurod6), mRNA.
10852019	Nfatc2	nuclear factor of activated T-cells, cytoplasmic, calcineurin-dependent 2 (Nfatc2), mRNA.
10826918	Nfkb1	similar to Nuclear factor NF-kappa-B p105 subunit gene:ENSRNOG00000023258
10935047	Ngfrap1	nerve growth factor receptor (TNFRSF16) associated protein 1 (Ngfrap1), mRNA.
10732649	Nkx2-5	NK2 transcription factor related, locus 5 (Drosophila) (Nkx2-5), mRNA.
10744600	Nlrp1a	51 kDa protein gene:ENSRNOG00000023143
10732257	Nme3	non-metastatic cells 3, protein expressed in (Nme3), mRNA.
10807174	Nol3	nucleolar protein 3 (apoptosis repressor with CARD domain) (Nol3), mRNA.
10864314	Npap60	nuclear pore associated protein, mRNA (cDNA clone MGC:93910 IMAGE:7112814), complete cds.
10749523	Nptx1	neuronal pentraxin 1 (Nptx1), mRNA.
10751328	Nr1i2	nuclear receptor subfamily 1, group I, member 2 (Nr1i2), mRNA.
10920461	Nradd	neurotrophin receptor associated death domain (Nradd), mRNA.
10875331	Nsmaf	neutral sphingomyelinase (N-SMase) activation associated factor (Nsmaf), mRNA.
10763889	Nuak2	NUAK family, SNF1-like kinase, 2 (Nuak2), mRNA.
10887667	Nucks1	nuclear casein kinase and cyclin-dependent kinase substrate 1 (Nucks1), mRNA.
10857752	Ogg1	8-oxoguanine DNA-glycosylase 1 (Ogg1), nuclear gene encoding mitochondrial protein, mRNA.
10759383	P2rx2	purinergic receptor P2X, ligand-gated ion channel, 2 (P2rx2), mRNA.
10899826	Pa2g4	proliferation-associated 2G4 (Pa2g4), mRNA.
10872790	Pafah2	platelet-activating factor acetylhydrolase 2 (Pafah2), mRNA.
10874539	Pank4	pantothenate kinase 4 (Pank4), mRNA.
10895337	Pawr	PRKC, apoptosis, WT1, regulator (Pawr), mRNA.
10715575	Pax2	paired box gene 2 (Pax2), mRNA.
10716136	Pdcd4	programmed cell death 4 (Pdcd4), mRNA.
10921190	Pdcd6ip	programmed cell death 6 interacting protein (Pdcd6ip), mRNA.
10846694	Pde1a	phosphodiesterase 1A, calmodulin-dependent (Pde1a), mRNA.
10862361	Pdia4	protein disulfide isomerase associated 4 (Pdia4), mRNA.
10872467	Pef1	penta-EF hand domain containing 1 (Pef1), mRNA.
10901111	Pef1	penta-EF hand domain containing 1 (Pef1), mRNA.
10701913	Perp	PERP, TP53 apoptosis effector (Perp), mRNA.
10895406	Phlda1	pleckstrin homology-like domain, family A, member 1 (Phlda1), mRNA.
10859397	Pik3c2g	phosphatidylinositol 3-kinase, C2 domain containing, gamma polypeptide (Pik3c2g), mRNA.
10800603	Pik3c3	phosphoinositide-3-kinase, class 3 (Pik3c3), mRNA.
10814726	Pik3ca|LOC68559	phosphatidylinositol 3-kinase, catalytic, alpha polypeptide, mRNA (cDNA clone IMAGE:7381259), complete cds.
10889607	Pik3cg	phosphoinositide-3-kinase, catalytic, gamma polypeptide (Pik3cg), mRNA.
10908265	Pin1	protein (peptidyl-prolyl cis/trans isomerase) NIMA-interacting 1 (Pin1), mRNA.
10751295	Pla1a	phospholipase A1 member A (Pla1a), mRNA.
10773708	Pla2g3	phospholipase A2, group III (Pla2g3), mRNA.
10859438	Plekha5	phosphoinositol 3-phosphate-binding protein-2-like protein mRNA, complete cds.
10744182	Polr2a	similar to DNA-directed RNA polymerase II largest subunit gene:ENSRNOG00000028834
10757674	Pom121	nuclear pore membrane protein 121 (Pom121), mRNA.
10785732	Pou4f1	similar to POU domain, class 4, transcription factor 1 gene:ENSRNOG00000009669
10807019	Pou4f2	similar to POU domain, class 4, transcription factor 2 gene:ENSRNOG00000012167
10804480	Ppic	peptidylprolyl isomerase C (Ppic), mRNA.
10926068	Ppid|RGD156014	peptidylprolyl isomerase D (cyclophilin D) (Ppid), mRNA.
10786163	Ppif	peptidylprolyl isomerase F (cyclophilin F) (Ppif), nuclear gene encoding mitochondrial protein, mRNA.
10804225	Ppp2r2b	protein phosphatase 2 (formerly 2A), regulatory subunit B (PR 52), beta isoform (Ppp2r2b), mRNA.
10770721	Ppp2r5a	protein phosphatase 2, regulatory subunit B (B56), alpha isoform (Ppp2r5a), mRNA.
10729673	Prkg1	protein kinase, cGMP-dependent, type 1 (Prkg1), mRNA.
10729689	Prkg1	protein kinase, cGMP-dependent, type 1 (Prkg1), mRNA.
10885808	Psen1	presenilin 1 (Psen1), mRNA.
10770298	Psen2	presenilin 2 (Psen2), mRNA.
10720658	Psenen	presenilin enhancer 2 homolog (C. elegans) (Psenen), mRNA.
10924876	Psmd1	proteasome (prosome, macropain) 26S subunit, non-ATPase, 1 (Psmd1), mRNA.
10910204	Pstpip1	proline-serine-threonine phosphatase-interacting protein 1 (Pstpip1), mRNA.
10714832	Pten	phosphatase and tensin homolog (Pten), mRNA.
10852474	Ptk6	PTK6 protein tyrosine kinase 6 (Ptk6), mRNA.
10902140	Ptprq	protein tyrosine phosphatase, receptor type, Q (Ptprq), mRNA.
10726108	Pycard	PYD and CARD domain containing (Pycard), mRNA.
10834945	Rapgef1	31 kDa protein gene:ENSRNOG00000014316
10712928	Rbm4b	RNA binding motif protein 4B (Rbm4b), mRNA.
10727634	Rbm4b|Rbm4	zinc responsive protein ZD7, mRNA (cDNA clone IMAGE:7301680), complete cds.
10867163	Rbpsuh	recombining binding protein suppressor of hairless (Drosophila) (Rbpsuh), mRNA.
10713089	Rela	v-rel reticuloendotheliosis viral oncogene homolog A (avian) (Rela), mRNA.
10864918	Ret	ret proto-oncogene (Ret), transcript variant 1, mRNA.
10851492	RGD1303142	oxidative stress responsive gene (RGD1303142), mRNA.
10894842	RGD1305457	similar to RIKEN cDNA 1700023M03, mRNA (cDNA clone MGC:108928 IMAGE:7382451), complete cds.
10701949	RGD1306565	similar to mitogen activated protein kinase kinase kinase 5 gene:ENSRNOG00000031700
10731738	RGD1310686	similar to chromosome 16 open reading frame 5, mRNA (cDNA clone MGC:94589 IMAGE:7190340), complete cds.
10834632	GD1311501|Adamts	similar to chromosome 9 open reading frame 7 (RGD1311501), mRNA.
10888917	RGD1311605	similar to apoptosis related protein APR-3 isoform 1 gene:ENSRNOG00000006326
10837125	RGD1564319	cDNA clone MGC:188637 IMAGE:7455398, complete cds.
10926390	RGD620382	Nucleoside 2-deoxyribosyltransferase domain containing protein RGD620382 (RGD620382), mRNA.
10783964	Ripk3	receptor-interacting serine-threonine kinase 3 (Ripk3), mRNA.
10763897	Ripk5	receptor interacting protein kinase 5 (Ripk5), mRNA.
10910754	Rpl4	ribosomal protein L4 (Rpl4), mRNA.
10704242	Rps5	ribosomal protein S5 (Rps5), mRNA.
10703144	Rps6ka2	similar to Ribosomal protein S6 kinase alpha 2 (S6K-alpha 2) (90 kDa ribosomal protein S6 kinase 2) (p90-RSK 2) (Ribosomal S6 kinase 3) (RSK-3) (pp90RSK3) (Protein-tyrosine kinase Mpk-9) (MAP kin
10755619	Rtn4r	reticulon 4 receptor (Rtn4r), mRNA.
10933716	Sat1	spermidine/spermine N1-acetyl transferase 1 (Sat1), mRNA.
10758137	Scarb1	scavenger receptor class B, member 1 (Scarb1), mRNA.
10771669	Sdad1	SDA1 domain containing 1 (Sdad1), mRNA.
10769807	Sdhc	succinate dehydrogenase complex, subunit C, integral membrane protein (Sdhc), nuclear gene encoding mitochondrial protein, mRNA.
10851502	Serinc3	serine incorporator 3 (Serinc3), mRNA.
10763375	Serpinb10	serine (or cysteine) peptidase inhibitor, clade B (ovalbumin), member 10 (Serpinb10), mRNA.
10872572	Sfn	similar to 14-3-3 protein sigma gene:ENSRNOG00000033153
10798610	Sfrp4	secreted frizzled-related protein 4 (Sfrp4), mRNA.
10784346	Sgcg	sarcoglycan, gamma (dystrophin-associated glycoprotein) (Sgcg), mRNA.
10937962	Sh3kbp1	SH3-domain kinase binding protein 1 (Sh3kbp1), mRNA.
10791406	Sh3md2	putative scaffolding protein POSH (Sh3md2), mRNA.
10887506	Siva1	SIVA1, apoptosis-inducing factor (Siva1), mRNA.
10792441	Slc20a2	solute carrier family 20, member 2 (Slc20a2), mRNA.
10921916	Slc25a27	solute carrier family 25, member 27 (Slc25a27), nuclear gene encoding mitochondrial protein, mRNA.
10736802	Slfn3	schlafen 3 (Slfn3), mRNA.
10715962	Slk	STE20-like kinase (yeast) (Slk), mRNA.
10805165	Smad4	MAD homolog 4 (Drosophila) (Smad4), mRNA.
10797127	Smad5	MAD homolog 5 (Drosophila) (Smad5), mRNA.
10714537	Smarca2	SWI/SNF related, matrix associated, actin dependent regulator of chromatin, subfamily a, member 2 (Smarca2), mRNA.
10908482	Smarca4	similar to SWI/SNF-related matrix-associated actin-dependent regulator of chromatin a4 gene:ENSRNOG00000009271
10709575	Smpd1	sphingomyelin phosphodiesterase 1, acid lysosomal (Smpd1), mRNA.
10842465	Snai1	snail homolog 1 (Drosophila) (Snai1), mRNA.
10862820	Snca	synuclein, alpha (Snca), mRNA.
10914061	Snrk	SNF-related serine/threonine-protein kinase gene:ENSRNOG00000004050
10939168	Snrk|LOC367880	SNF related kinase (Snrk), mRNA.
10745107	Spag5	sperm associated antigen 5 (Spag5), mRNA.

10837520	St13	suppression of tumorigenicity 13 (St13), mRNA.
10889719	St13	suppression of tumorigenicity 13 (St13), mRNA.
10905589	St13	suppression of tumorigenicity 13 (St13), mRNA.
10866780	St8sia1	ST8 alpha-N-acetyl-neuraminide alpha-2,8-sialyltransferase 1 (St8sia1), mRNA.
10863559	Stambp	Stam binding protein (Stambp), mRNA.
10732644	Stc2	stanniocalcin 2 (Stc2), mRNA.
10931147	Steap3	STEAP family member 3 (Steap3), mRNA.
10903246	Stk3	serine/threonine kinase 3 (STE20 homolog, yeast) (Stk3), mRNA.
10874981	Sulf1	sulfatase 1 (Sulf1), mRNA.
10842086	Svs4	seminal vesicle secretory protein 4 (Svs4), mRNA.
10713160	Syvn1	similar to synoviolin 1 isoform a isoform 1 gene:ENSRNOG00000020950
10938952	Taf9b	TAF9B RNA polymerase II, TATA box binding protein (TBP)-associated factor (Taf9b), mRNA.
10762515	Taok3	TAO kinase 3 (Taok3), mRNA.
10762378	Tbx3	T-box 3 (Tbx3), mRNA.
10899125	Tegt	testis enhanced gene transcript (Tegt), mRNA.
10869946	Tek	endothelial-specific receptor tyrosine kinase (Tek), mRNA.
10717517	Tert	telomerase reverse transcriptase (Tert), mRNA.
10703744	Tfpt	TCF3 (E2A) fusion partner (Tfpt), mRNA.
10891303	Tgfb3	transforming growth factor, beta 3 (Tgfb3), mRNA.
10851350	Tgm2	transglutaminase 2, C polypeptide (Tgm2), mRNA.
10792216	Thap1	THAP domain containing, apoptosis associated protein 1 (Thap1), mRNA.
10881925	Thap3	THAP domain containing, apoptosis associated protein 3 (Thap3), mRNA.
10901231	Timp3	tissue inhibitor of metalloproteinase 3 (Timp3), mRNA.
10788238	Tlr3	toll-like receptor 3 (Tlr3), mRNA.
10815503	Tm4sf4	transmembrane 4 superfamily member 4 (Tm4sf4), mRNA.
10921117	Tmem158	transmembrane protein 158 (Tmem158), mRNA.
10868817	Tmod1	tropomodulin 1 (Tmod1), mRNA.
10828021	Tnf	tumor necrosis factor (TNF superfamily, member 2) (Tnf), mRNA.
10781321	Tnfrsf10b	tumor necrosis factor receptor superfamily, member 10b (Tnfrsf10b), mRNA.
10740869	Tnfrsf12a	tumor necrosis factor receptor superfamily, member 12a (Tnfrsf12a), mRNA.
10858967	Tnfrsf1a	tumor necrosis factor receptor superfamily, member 1a (Tnfrsf1a), mRNA.
10881424	Tnfrsf1b	tumor necrosis factor receptor superfamily, member 1b (Tnfrsf1b), mRNA.
10744171	Tnfsf12	tumor necrosis factor ligand superfamily member 12 (Tnfsf12), mRNA.
10810556	Tradd	similar to TNFRSF1A-associated via death domain gene:ENSRNOG00000015179
10830398	Traf3ip2	Traf3 interacting protein 2 (Traf3ip2), mRNA.
10838100	Traf6	Tnf receptor-associated factor 6 (Traf6), mRNA.
10731853	Trap1	TNF receptor-associated protein 1 (Trap1), mRNA.
10762701	Triap1|LOC68783[illegible]	TP53 regulated inhibitor of apoptosis 1 (Triap1), mRNA.
10850775	Trib3	tribbles homolog 3 (Drosophila) (Trib3), mRNA.
10867609	Trp53inp1	transformation related protein 53 inducible nuclear protein 1 (Trp53inp1), mRNA.
10755035	Trp63	transformation related protein 63 (Trp63), mRNA.
10722167	Tsg101	tumor susceptibility gene 101 (Tsg101), mRNA.
10738296	Tubg1	tubulin, gamma 1 (Tubg1), mRNA.
10925449	Twist2	twist homolog 2 (Drosophila) (Twist2), mRNA.
10877005	Txn1	thioredoxin 1 (Txn1), mRNA.
10817552	Txnip	thioredoxin interacting protein (Txnip), mRNA.
10833013	Unc5b	unc-5 homolog B (C. elegans) (Unc5b), mRNA.
10819469	Unc5c	unc-5 homolog C (C. elegans) (Unc5c), mRNA.
10747633	Vat1	vesicle amine transport protein 1 homolog (T californica) (Vat1), mRNA.
10733438	Vdac1	Voltage-dependent anion-selective channel protein 1 gene:ENSRNOG00000006375
10804519	Vdac1	voltage-dependent anion channel 1 (Vdac1), mRNA.
10940030	Vdac1	voltage-dependent anion channel 1 (Vdac1), mRNA.
10842043	Wisp2	WNT1 inducible signaling pathway protein 2 (Wisp2), mRNA.
10738707	Wnt3	wingless-related MMTV integration site 3 (Wnt3), mRNA.
10914811	Yap1	yes-associated protein 1 (Yap1), mRNA.
10889360	Ywhaq	tyrosine 3-monooxygenase/tryptophan 5-monooxygenase activation protein, theta polypeptide (Ywhaq), mRNA.
10764523	Zc3h15	zinc finger CCCH-type containing 15 (Zc3h15), mRNA.
10837266	Zc3h15	zinc finger CCCH-type containing 15 (Zc3h15), mRNA.
10754629	Zfp148	zinc finger protein 148 (Zfp148), mRNA.
10813949	Zfp622	Zinc finger protein 622 gene:ENSRNOG00000010589
10813951	Zfp622	Zinc finger protein 622 gene:ENSRNOG00000010589
10813953	Zfp622	zinc finger protein 622 (Zfp622), mRNA.
10927209	Zfp622	zinc finger protein 622 (Zfp622), mRNA.
10814717	Zmat3	zinc finger, matrin type 3 (Zmat3), mRNA.

ıase-activated protein kinase 1c) (MAPKAPK1C)... isoform 6 gene:ENSRNOG00000013194
